# Comparison of factors of the subjective psychological condition of hospitalized individuals with schizophrenia spectrum and other psychotic disorders, or affective disorders: associations between illness perception, quality of life, affective and anxiety symptom severity, and readiness to change

**DOI:** 10.3389/fpsyg.2026.1829834

**Published:** 2026-10-02

**Authors:** Kilian Lommer, Timo Schurr, Kristina Stürz, Bernhard Holzner, Alex Hofer

**Affiliations:** 1 Division of Psychiatry I, Department of Psychiatry, Psychotherapy, Psychosomatics and Medical Psychology, Medical University Innsbruck, Innsbruck, Austria; 2 Department of Health Psychology, School of Population Health, RCSI University of Medicine and Health Sciences, Dublin, Ireland

**Keywords:** affective disorders, illness perception, psychotic disorders, quality of life, readiness to change, schizophrenia

## Abstract

Illness perception in mental health disorders can affect prognosis and well-being. According to the Transtheoretical Model of Behavior Change, mental illness involves repetitive behavioral patterns that gradually change throughout the therapeutic process, during which progressing to stages of higher readiness to change is considered advantageous. The current study examined correlations between illness perception, readiness to change, quality of life, and the severity of depression and anxiety in hospitalized individuals with psychotic disorders or affective disorders. Data from 110 individuals with schizophrenia spectrum and other psychotic disorders (ICD-10: F20–29, F2* group) and 261 individuals with affective disorders (ICD-10: F30–39, F3* group) were analyzed. Illness perception, readiness to change, quality of life, and depression and anxiety symptom severity were assessed using the Brief Illness Perception Questionnaire, the “Fragebogen zur Erfassung der Veränderungsbereitschaft” (Readiness for Change Questionnaire), the brief version of the World Health Organization’s Quality of Life assessment tool, the Beck Depression Inventory and the Beck Anxiety Inventory. Various illness perception dimensions were associated with quality of life and depression scores in both groups, and with anxiety scores in the F3* group. The advantageous readiness to change stage of *Action* correlated with the *Treatment Control* and *Coherence* dimensions of illness perception in both groups. Regression analyses revealed that *Physical Quality of Life* and depression scores were the strongest predictors of several dimensions of illness perception. These findings underscore the importance of illness perception in mental health disorders. Understanding one’s illness and its treatment emerged as a significant aspect regarding advantageous stages of change across diagnostic groups. Physical quality of life and depressive symptoms should be addressed in the treatment of both psychotic and affective disorders.

## 1 Introduction

Illness perceptions refer to the cognitive representations that individuals with an illness hold of their condition. According to Leventhal’s et al. (2003) Common Sense Model of Self-Regulation, individuals form mental representations of their illness to gain an individual understanding of it and to help them cope. These include beliefs and expectations relating to the causes, identity, progression and treatment options of the illness, as well as its consequences for patients’ lives. It has been suggested that a patient’s understanding of their illness, particularly in the early stages of diagnosis, is shaped by society’s perception of that illness. This understanding then undergoes a dynamic process of re-evaluation and change as the patient gains experience and receives information about their condition (Petrie et al., 2008). While the perception of illness was initially studied in relation to somatic diseases (Dempster et al., 2015), it has subsequently been investigated in relation to mental health conditions as well (Cannon et al., 2022). It has been shown that a patient’s beliefs influence their illness-related behavior. For example, a qualitative study on patients’ belief about bipolar disorder found that viewing one’s illness as an acute condition may hinder adherence to long-term pharmacological treatment after initial symptom reduction, thereby jeopardizing the improvements achieved through treatment in the acute phase of the illness (Clatworthy et al., 2007).

Empirical investigations have shown that aspects of illness perception are associated with severity and outcome measures in both somatic and mental illnesses (Broadbent et al., 2015; Dempster et al., 2015; Etain et al., 2018; Mavroeides and Koutra, 2021; Cannon et al., 2022). Indeed, a meta-analysis of studies investigating the relationship between illness perception and outcome measures in individuals with various mental health disorders revealed medium-to-large associations between aspects of illness representations and better or worse outcomes, respectively (Cannon et al., 2022), which has been reported similarly in a review of studies with a narrower focus on illness perception in depression (Mavroeides and Koutra, 2021). A number of studies on illness perception in patients experiencing schizophrenia and other psychotic disorders reported similar association patterns between aspects of illness perception and outcome measures (Lobban et al., 2004; Watson et al., 2006; Gómez-de-Regil, 2015). Illness perception dimensions have been reported to moderate between insight and depression (Cavelti et al., 2012) and predict quality of life (Theodore et al., 2012) in patients experiencing psychotic disorders. The authors of the previously mentioned meta-analysis across diagnoses noted that the available data did not allow for a formal comparison of associations between illness perception dimensions and clinical outcomes across diagnostic groups, e.g., between depression and schizophrenia (Cannon et al., 2022). Possible between-group differences of the relationship of illness perception and outcome measures have not been thoroughly researched presently.

The Transtheoretical Model of Behavior Change as proposed by Prochaska and DiClemente (1983) considers health-related behaviors to be subject to stages of change over time. It assumes that beneficial behavioral change takes place by moving along a number of defined stages, including precontemplation, contemplation, preparation, action, and maintenance. Beyond its primary application of describing processes of behavioral change around habits relevant for physical well-being, such as smoking or alcohol consumption, it has since been applied to psychotherapy-related processes in the treatment of mental illnesses (Prochaska and Norcross, 2001), defining common processes of change taking place across different therapeutic approaches. Also, a meta-analysis has shown the stages of change to reliably predict psychotherapy outcomes across various diagnoses, demonstrating a medium effect size (Krebs et al., 2018). A recent study of individuals with anorexia nervosa found that those in the Action stage had a stronger belief in the efficacy of treatment and were more concerned about and more aware of their illness (Neu et al., 2025), which we sought to reproduce in different diagnostic cohorts.

In the context of these findings, the current study aimed to explore the associations between illness perception, readiness to change, quality of life, and the severity of depressive and anxious symptoms in hospitalized individuals with schizophrenia spectrum and other psychotic disorders, or affective disorders. We hypothesized that there would be significant correlations between dimensions of illness perception, symptom severity, and quality of life scores. Regarding the stages of change, we expected the advanced stage of *Action* to be associated with a stronger belief in the efficacy of treatment, greater concern about the illness, and a better understanding of it.

## 2 Materials and methods

### 2.1 Participants

The study included individuals diagnosed with schizophrenia spectrum and other psychotic disorders (ICD-10: F20-29, F2* group), or affective disorders (ICD-10: F30-39; F3* group) > 18 years of age who were admitted for inpatient treatment at the Department of Psychiatry, Psychotherapy, Psychosomatics and Medical Psychology of the Medical University Innsbruck on two inpatient units. One unit focused explicitly on short-term treatment and stabilization, and the other specialized in the treatment of psychotic and affective disorders. The inpatient treatment oftentimes included adaptations or transient expansion of pharmacological treatment, occupational therapy, physical therapy and activities in a group setting, in many cases short-term individual psychotherapeutic interventions, and on the latter ward psychotherapeutic groups. Data on medication load and therapies employed were not recorded. As part of the routine data acquisition process, patients were asked to complete several self-administered Likert-style questionnaires at the beginning of inpatient treatment, which were collected digitally on the Computer-based Health Evaluation System (Holzner et al., 2012). The primary aim of this data collection was to support clinical decision-making.

### 2.2 Measures

The Brief Illness Perception Questionnaire (BIPQ) (Broadbent et al., 2006) is a nine-item scale developed for the rapid assessment of illness perception. Item 1 through 8 represent the following dimensions of illness perception: (1) *Consequences* (“How much does your illness affect your life?”), (2) *Timeline* (“How long do you think your illness will continue?”), (3) *Personal Control* (“How much control do you feel you have over your illness?”), (4) *Treatment Control* (“How much do you think your treatment can help your illness?”), (5) *Identity* (“How much do you experience symptoms from your illness?”), (6) *Illness Concern* (“How concerned are you about your illness?”), (7) *Coherence* of the person’s understanding of the illness (“How well do you feel you understand your illness?”), and (8) *Emotional representation* (“How much does your illness affect you emotionally?”). Items are to be rated on a scale of 1–10, with higher scores indicating greater approval. Some authors distinguish between threat-related aspects (*Consequences, Timeline, Identity, Illness Concern,* and *Emotional representation*) and protective aspects of illness perception (*Personal control, Treatment control*, and *Coherence*) (Cannon et al., 2022), while others group illness perception dimensions into cognitive (*Consequences, Timeline, Personal control, Treatment control*, and *Identity*), emotional (*Illness Concern* and *Emotional representation*), and comprehensibility (*Coherence*) domains (Gómez-de-Regil, 2014). In the presented study, we analyzed the BIPQ on a single-item level instead of the mentioned domain-based approaches, but the latter understanding may be employed to inform the interpretation of results. The ninth BIPQ item asks participants to list the three factors that they subjectively perceive to be the most important causal factors of the illness. We did not include the latter, as the item’s format is not conducive to quantitative analysis.

Quality of life was assessed using the Brief Version of the World Health Organization Quality of Life Assessment (WHOQOL-BREF) (The WHOQOL Group, 1998), which has been shown to exhibit favorable psychometric properties (The WHOQOL Group, 1998). The WHOQOL-BREF is a 26-item questionnaire based on a longer version of the same scale and assesses quality of life in the following domains: global quality of life, physical health, psychological health, social relationships, and environment. Each question is rated on a 5-point scale, domain scores are transformed to lie between 0 and 100. Higher ratings represent a higher reported quality of life.

The Beck Depression Inventory (BDI) is a very well-established scale for the assessment and quantification of depressive symptoms. We made use of the revised BDI-II, which consists of 21 items to be rated by participants themselves on four levels (Beck et al., 1996) and whose German language translation has been shown to be a valid tool in the assessment of depressive psychopathology (Kühner et al., 2007).

The Beck Anxiety Inventory (BAI) is a similarly widely established scale developed for measuring the severity of anxiety that, similarly to the BDI, consists of 21 items rated by participants on four levels (Beck et al., 1988). A recent validation study of the German language version has found it to be a brief, user-friendly, unidimensional tool (Geissner and Huetteroth, 2018).

The “Fragebogen zur Erfassung der Veränderungsbereitschaft” (FEVER, “Readiness for Change Questionnaire”) (Hasler et al., 2003) is the German language version of the University of Rhode Island Change Assessment Scale (McConnaughy et al., 1989). The scale consists of 24 items to be rated in a five-level design, reflecting the stages of behavioral change as conceptualized in Prochaska’s Transtheoretical Model, namely *Precontemplation*, *Contemplation*, and *Action.* Due to practical and statistical considerations, the two stages of Preparation and Maintenance originally included in the model have been dropped by the authors of the German translation (Hasler et al., 2003) and are therefore not reflected in our data.

The study was approved by the Ethics Committee of the Medical University of Innsbruck (approval no. 1128/2020).

### 2.3 Statistical methods

Statistical analyses were carried out using R version 4.4.1 (R Core Team, 2016), with the corrplot (v0.92) package (Wei and Simko, 2021) also being used. The statistical significance level was set to *α* = 0.05, all hypothesis tests were two-sided. To assess the robustness of findings to multiple testing, Benjamini–Hochberg false discovery rate (FDR) adjusted *p*-values were additionally calculated as a sensitivity analysis within each diagnostic group and predictor domain.

Analyses were conducted separately for patients with F2* and F3* diagnoses. Missing data were not imputed. First, exploratory Spearman correlation coefficients were computed between all questionnaire measures, visualized using heatmaps. Complete results including non-significant findings are reported in the Supplementary materials.

For the regression analyses, model-specific complete-case samples were used; that is, a participant was included in a given model only if data were available for the respective BIPQ outcome and all predictors included in that model.

Multiple linear regression analyses were subsequently performed separately for each of the eight BIPQ domains: Consequences, Timeline, Personal Control, Treatment Control, Identity, Illness Concern, Coherence, and Emotional Representation. For each BIPQ domain and diagnostic group, three prespecified predictor-domain models were estimated. In the first model, the three FEVER dimension scores: Precontemplation, Contemplation, and Action were entered simultaneously as predictors.

These models directly examined the associations between dimensional stages-of-change scores and the respective illness-perception domain. In the second model, the four WHOQOL-BREF domains: Physical, Psychological, Social, and Environment were entered simultaneously. In the third model, BDI-II and BAI total scores were entered simultaneously.

For each regression model, unstandardized regression coefficients (*B*), standard errors (SE), 95% confidence intervals for *B*, standardized regression coefficients (*β*), 95% confidence intervals for *β*, and two-sided *p*-values were reported. Model fit was summarized using *R*^2^ and adjusted *R*^2^. Collinearity was assessed using variance inflation factors (VIF) and tolerance statistics.

To improve transparency regarding missing data, the number and percentage of missing observations were summarized for each study variable, questionnaire-block completeness patterns were described separately by diagnostic group, and the number of complete cases was reported for each regression model. In addition, completers and non-completers within each predictor domain were compared with respect to age, sex, mother tongue, and Nationality. Differences were primarily summarized using standardized mean differences to avoid introducing an additional large family of significance tests.

As an additional exploratory sensitivity analysis, models containing all nine predictor variables simultaneously; that is, the three FEVER dimensions, four WHOQOL-BREF domains, BDI-II total score, and BAI total score were estimated for each BIPQ outcome where the available complete-case sample permitted estimation.

The BIPQ total score displayed in the correlation figures was calculated by summing Consequences, Timeline, Identity, Illness Concern, and Emotional Representation in their original direction and reverse-scoring Personal Control, Treatment Control, and Illness Coherence as 10 minus the original score before summation. The resulting total score ranged from 0 to 80, with higher scores reflecting a more negative or threatening overall illness representation. The individual BIPQ domains used as outcomes in the regression analyses were retained in their original scoring direction.

Complete regression results, including model-specific sample sizes and both nominal and FDR-adjusted *p*-values, are provided in the Supplementary Tables.

## 3 Results

### 3.1 Sample

A total of 371 patients took part in this study, 110 of whom were in the F2* group (39.2% females, mean age 43.1 ± 14.3 years, mean time since first inpatient admission 7.5 ± 9.0 years). The F3* group comprised a further 261 people (42.6% females, mean age 47.4 ± 16.9 years, mean time since first inpatient admission 6.0 ± 9.7 years). 115 patients were treated on the ward specialized on the treatment of affective and psychotic disorders, while the remaining 256 were treated on the ward focusing on short-term treatment and stabilization.

### 3.2 Correlational analyses

The results of the correlation analyses after adjusting *p*-values for the false discovery rate (FDR) are shown in Figures 1, 2.

**Figure 1:**
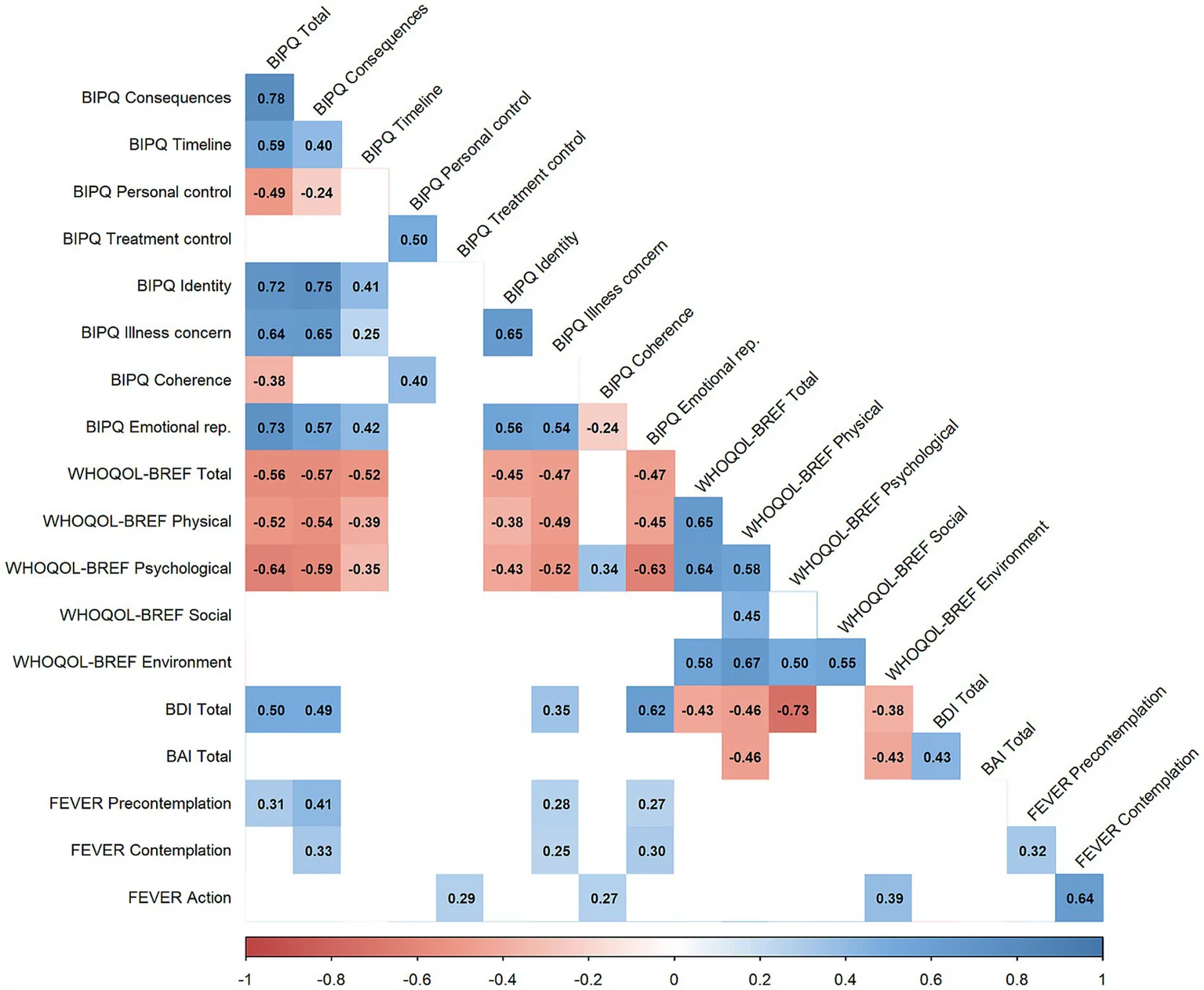
Significant (adjusted *p* < 0.05) correlations (Spearman’s rho) after Benjamini–Hochberg FDR correction in the F2* group. The color and intensity of the color indicate the direction and strength of the correlation. BIPQ, Brief Illness Perception Questionnaire; WHOQOL-BREF, World Health Organization Quality of Life Assessment, Brief Version; BDI, Beck Depression Inventory; BAI, Beck Anxiety Inventory; FEVER, Fragebogen zur Erfassung der Veränderungsbereitschaft (Readiness for Change Questionnaire).

**Figure 2:**
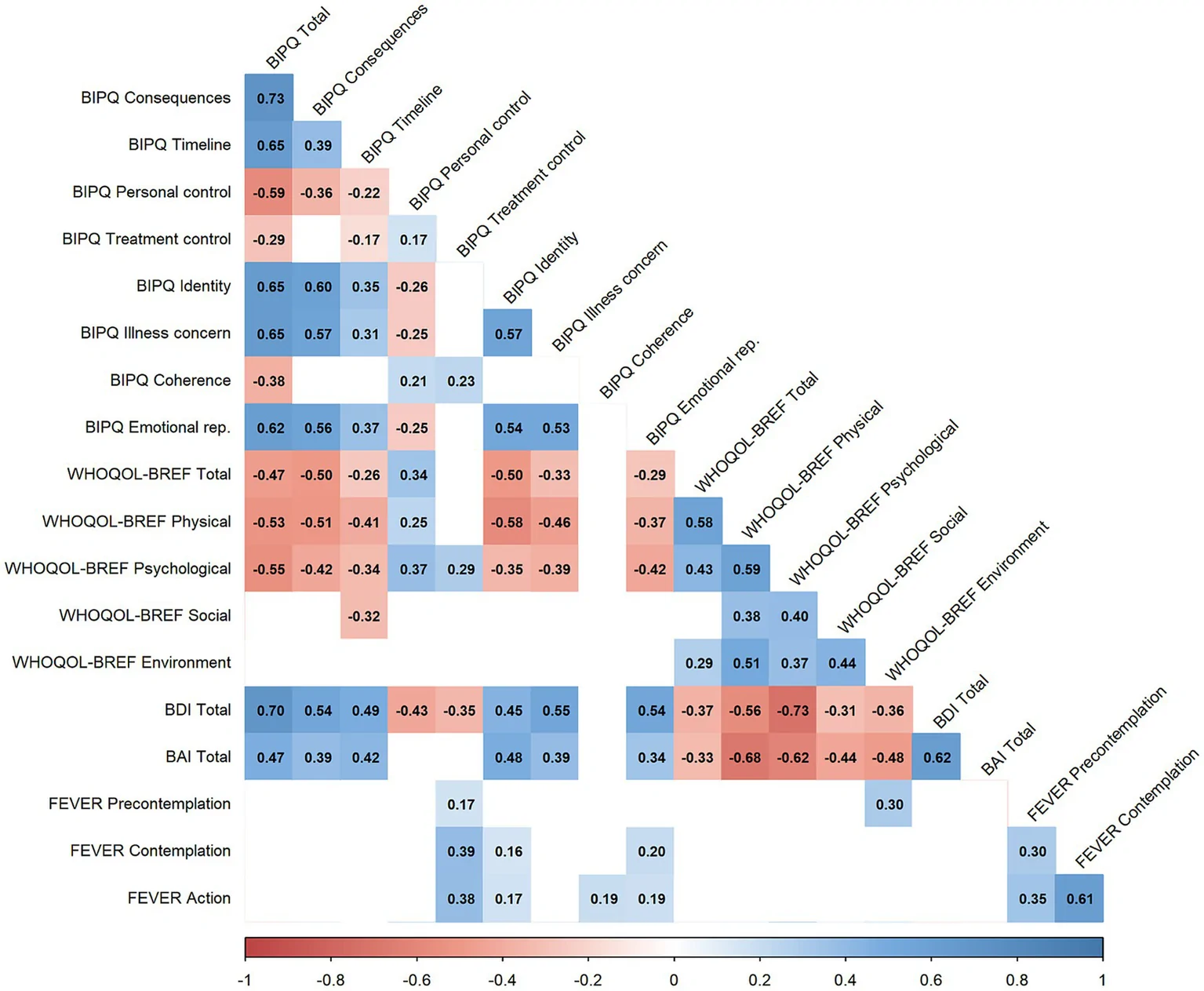
Significant (adjusted *p* < 0.05) correlations (Spearman’s *rho*) after Benjamini-Hochberg FDR correction in the F3* group. The color and intensity of the color indicate the direction and strength of the correlation. BIPQ, Brief Illness Perception Questionnaire; WHOQOL-BREF, World Health Organization Quality of Life Assessment, Brief Version; BDI, Beck Depression Inventory; BAI, Beck Anxiety Inventory; FEVER, Fragebogen zur Erfassung der Veränderungsbereitschaft (Readiness for Change Questionnaire).

Across both diagnostic groups, a large number of BIPQ items correlated significantly with the global quality of life and the physical and psychological subdomain scores of the WHOQOL-BREF. By contrast, illness perception appeared to be associated with social and environmental quality of life to a smaller degree.

Regarding anxiety as reflected by BAI scores, no item showed a significant correlation in the F2* group. In the F3* group, however, several BIPQ items were positively correlated with the BAI. Regarding depression, most BIPQ items significantly correlated with BDI scores in the F3* group and a number of items in the F2* group.

We also observed several correlations with the scores of the different stages of change as represented by the FEVER. In the F2* group, *Treatment control* and *Coherence* were significantly positively correlated with an advanced stage of *Action*. On the other hand, in the F3* group, *Treatment control*, Identity, *Coherence*, and *Emotional representation* were significantly positively correlated with an advanced stage of *Action*. No BIPQ item showed a significant negative correlation with the *Action* subscore. In both groups, only the *Action* subscore of the FEVER was positively correlated with *Coherence*.

### 3.3 Regression analyses

Further insights were provided by regression analyses with BIPQ dimensions as dependent variables, revealing significant connections not represented in the correlational analyses. Table 1 shows significant (*p* < 0.05) statistical predictions not already reflected in the correlational analysis. In the F2* group, the *Action* subscore of the FEVER was found to be a significant negative statistical predictor of the *Consequences* dimension of the BIPQ, when controlling for the other stages of change. Furthermore, *Social quality of life* of the WHOQOL-BREF was a significant statistical predictor of *Identity* when controlling for the other aspects of quality of life. *Environmental quality of life* was a significant statistical predictor of the BIPQ *Illness Concerns* item. In turn, regression analyses revealed only one significant statistical prediction beyond the significant correlations in the F3* group: *Environmental quality of life* was found to negatively predict the BIPQ item of *Personal control*. Note that these findings did not remain statistically significant after Benjamini–Hochberg adjustment of *p*-values and should be considered exploratory.

**Table 1:** Significant (*p* < 0.05) results of regression analyses with BIPQ dimensions as dependent variables beyond those reflected in the correlational analysis.

Group	Predictor	Outcome	*β*	95% confidence interval for *β* lower bound/upper bound		*p* FDR
F2*	*Action* subscore	*Consequences*	−0.31	−0.62	−0.01	0.210
F2*	*Social quality of life*	*Identity*	0.31	0.01	0.61	0.184
F2*	*Environmental quality of life*	*Illness Concerns*	0.34	0.01	0.67	0.187
F3*	*Environmental quality of life*	*Personal Control*	−0.27	−0.52	−0.03	0.113

*β* = standardized regression coefficient; *p* FDR values were adjusted using the Benjamini–Hochberg false discovery rate procedure within each diagnostic group and predictor block. Each regression used model-specific complete cases.

Table 2 presents the largest significant (*p* < 0.05) standardized associations within the specified model, especially those with *β* > 0.5, to determine which factors have the greatest predictive statistical value for illness perception dimensions. In the F2* group, *Physical quality of life* as measured by the WHOQOL-BREF proved to be a strong negative statistical predictor of illness perception in terms of *Timeline* and a moderately strong negative statistical predictor of *Illness Concern*. *Psychological quality of life* was a strong negative statistical predictor of *Emotional representation*. Additionally, BDI scores were strong negative statistical predictors of the illness perception aspects of *Coherence* and strong positive statistical predictors of *Emotional representation*. In the F3* group, mostly the same variables were identified as having the strongest predictive statistical value, albeit mostly for other aspects of illness perception. *Physical quality of life* was found to be a strong negative statistical predictor of *Identity* as well as a moderately strong negative statistical predictor of *Consequences*, *Timeline*, and *Illness Concern*. Furthermore, BDI scores were found to be strong positive statistical predictors of the illness perception dimensions of *Consequences* and *Emotional representation* and strong negative statistical predictors of *Treatment control*, as well as moderately strong positive statistical predictors of *Illness Concern*. Note that only some of these associations retained statistical significance after correcting *p*-values following the Benjamini–Hochberg procedure, among them particularly those predicted by the BDI in both groups and by physical quality of life in the F3* group.

**Table 2:** Significant (*p* < 0.05) results of regression analyses with BIPQ dimensions as dependent variables with the largest regression coefficients.

Group	Predictor	Outcome	*β*	95% confidence interval for *β* lower bound/upper bound		*p* FDR
F2*	*Physical quality of life*	*Timeline*	−0.53	−0.95	−0.12	0.078
F2*	*Physical quality of life*	*Illness concern*	−0.49	−0.82	−0.15	0.053
F2*	*Psychological quality of life*	*Emotional representation*	−0.61	−0.90	−0.32	0.004
F2*	BDI	*Coherence*	−0.55	−0.88	−0.22	0.016
F2*	BDI	*Emotional representation*	0.70	0.41	0.99	<0.001
F3*	*Physical quality of life*	*Identity*	−0.66	−0.91	−0.42	<0.001
F3*	*Physical quality of life*	*Consequences*	−0.47	−0.73	−0.22	0.006
F3*	*Physical quality of life*	*Timeline*	−0.40	−0.67	−0.14	0.023
F3*	*Physical quality of life*	*Illness Concern*	−0.44	−0.70	−0.18	0.014
F3*	BDI	*Consequences*	0.54	0.22	0.86	0.009
F3*	BDI	*Treatment Control*	−0.62	−0.96	−0.28	0.009
F3*	BDI	*Emotional representation*	0.53	0.21	0.86	0.009
F3*	BDI	*Illness Concern*	0.43	0.10	0.76	0.050

*β* = standardized regression coefficient; *p* FDR values were adjusted using the Benjamini–Hochberg false discovery rate procedure within each diagnostic group and predictor block. Each regression used model-specific complete cases.

## 4 Discussion

### 4.1 Illness perception, symptom scores, and quality of life

Our findings generally corroborate the association between illness perception dimensions and measures of symptom severity and quality of life, as previously reported in reviews of illness perception across various mental health disorders (Cannon et al., 2022). We also confirmed that protective and threat-related illness perception dimensions are distinguished by their opposite associations with these measures (Cannon et al., 2022), as the BIPQ items relating to *Personal control, Treatment control*, and *Coherence* (protective dimensions) showed an opposite correlation compared to the remaining threat-related dimensions.

Our findings in the F2* group are generally consistent with previous reports on the role of illness perception in individuals with non-affective psychosis, as well as its associations with depression and anxiety measures (Watson et al., 2006; Gómez-de-Regil, 2015). For example, Gómez-de-Regil (2015) reported an association between the cognitive and emotional aspects of illness perception and depression, which aligns with our findings. Additionally, we found that the *Coherence* dimension of the BIPQ was negatively correlated with the severity of depression, which again corroborates earlier research (Lobban et al., 2004). However, this did not retain significance after correcting for FDR. Our data only partially reflect the association with anxiety mentioned in the study by Gómez-de-Regil (2015) since the *Consequences* dimension of the BIPQ was the only one that correlated with BAI scores in our sample, the significance of which did similarly not persist beyond FDR correction. Unlike our study sample, Gómez-de-Regil (2015) only examined non-hospitalized patients with mild symptoms. It is unclear to what extent the difference in setting or a potential difference in symptom severity may explain the discrepancy because no formal instrument for assessing psychotic symptoms was used in our study. While illness perception dimensions appear to be associated with symptom severity in several domains, this observation should not be considered a comprehensive understanding, as it is most likely part of a broader interplay between a wider scope of psychological and psychosocial factors. Exemplifying this, insight may play a crucial role in the interplay of the discussed dimensions due to the nature of psychotic disorders, as illness perception dimensions have been shown to mediate the relationship between insight and depressive symptoms (Cavelti et al., 2012).

In the F3* group, our findings largely mirrored those of a systematic review of illness perception in depression (Mavroeides and Koutra, 2021), which showed that unfavorable representations of *Identity, Control, Consequences, Coherence*, and chronic *Timeline* were associated with poorer depression outcomes. Our findings, notably in a sample across affective illnesses, differed in that we additionally found *Illness Concern* and *Emotional Representation* to be associated with more severe depression, but we did not find higher *Coherence* to be associated with lower depression severity. Similar to our findings, *Illness concern* has been shown to hold significance in bipolar disorder, albeit in regard to greater number of relapses and to psychosocial recovery (Lobban et al., 2013; Dodd et al., 2017). Regarding *Emotional Representation*, the literature provides conflicting findings. Mavroeides and Koutra (2021) found that the majority of publications did not show significant associations with depression outcomes, but, where significant, they are in line with our findings of greater *Emotional Representation* being connected to poorer depression severity (Lu et al., 2014; Glattacker et al., 2018). Mavroeides and Koutra (2021) also observed that while *Coherence* was a relatively under-investigated dimension, it was mostly associated with more advantageous depression and functional scores (Glattacker et al., 2013; Lu et al., 2014). This is contradicted by our finding of a lack of significant associations of *Coherence* with depression and quality of life scores in a sample across affective disorders. In cohorts of individuals experiencing bipolar disorder, Lobban et al. (2013) likewise reported no significant association between the *Coherence* dimension and mood, while Dodd et al. (2017) reported no significant association with subjective psychosocial recovery. It is therefore thinkable that illness perception, in the form of individuals’ perception of how thorough an understanding of their illness they have and to what degree they are concerned by and feel themselves to be emotionally affected by their illness, might have different effects on the intensity of affective symptoms and psychosocial functioning depending on whether the underlying condition is unipolar or bipolar depression.

To expand on the point of differences between diagnostic groups, we initially found the *Coherence* dimension to be negatively correlated with depression scores in the F2* group, which did not retain significance after FDR correction, but found no such association in the F3* group. Next to contradicting the findings of Mavroeides and Koutra (2021), who reported lower depression scores to be connected to a better understanding of one’s illness in depression, this also contrasts the report of more severe depression scores and poorer quality of life being associated with greater *Coherence* in schizophrenia (Lobban et al., 2004). While it does indeed appear that the role of illness *Coherence* may be disease dependent (Mavroeides and Koutra, 2021), further research is needed to attain clearer perspectives on the directionality and between-diagnosis differences of this role.

Regarding the severity of symptoms of anxiety in depression, the review identified greater *Consequences, Timeline, Identity,* and *Emotional representation* as being correlated with more severe anxiety. Meanwhile, *Personal Control* and *Treatment* Control were found to be associated with less severe anxiety (Mavroeides and Koutra, 2021). We found the same dimensions, as well as *Illness Concern*, to be associated with greater levels of anxiety. In our data, however, no illness perception dimensions were significantly correlated with less severe anxiety. In affective disorders, illness perception has been identified as a significant factor in seeking treatment (O’Mahen et al., 2009) and adhering to therapeutic recommendations (Clatworthy et al., 2007; Averous et al., 2018). Furthermore, illness perception dimensions in bipolar disorder were associated with relapse rates, with greater *Consequences* and *Emotional representation* being associated with higher and *Identity* being associated with lower likelihood of relapse (Lobban et al., 2013). Also, a summary score of illness perception dimensions across the BIPQ has been shown to be a predictor of lower personal recovery, i.e., the ability to engage in meaningful activities (Dodd et al., 2017). These findings reflect the clinical, prognostic, and subjective relevance of illness perception in individuals with affective disorders.

In terms of quality of life, the cognitive and emotional, rather than comprehensibility, aspects of illness perception, have previously been identified as factors that mediate the relationship between residual psychotic symptoms and quality of life (Gómez-de-Regil et al., 2014). This is in line with the findings of Theodore et al., who reported no significant correlation between *Coherence* and quality of life in a sample of patients experiencing early psychosis (Theodore et al., 2012). Although we did not perform a mediation analysis and analyzed the BIPQ findings by single item rather than by domain, our results in contrast suggest a positive correlation between the *Coherence* dimension, which reflects the comprehensibility domain, and psychological quality of life, while the other dimensions of quality of life were not significantly correlated. Lobban et al. (2004) reported a significant correlation with the opposite direction in a sample of individuals experiencing schizophrenia, suggesting that greater illness understanding is correlated with poorer quality of life. The discrepancies between findings may be explained by differences between samples, as our sample included in-patient participants experiencing various psychotic disorders, while Theodore et al. included mostly outpatients and Lobban et al. (2004) and Gómez-de-Regil et al. (2014) included only patients with a diagnosis of schizophrenia. It is thinkable the relationship between illness comprehensibility and quality of life could be influenced by the diagnosis and by aspects of the broader psychological and psychosocial context.

To further this point, it should be noted that quality of life in severe mental illness is influenced by a wide range of factors beyond those considered in the current investigation, among them social and environmental context, stigma and self-stigma, and service setting. Due to the nature of the data collection process, which was a routine feature of the process of admission into clinical care, quality of life was assessed at the beginning of an in-patient treatment stay. This is a treatment setting that oftentimes follows an acute crisis or exacerbation of pre-existing symptoms, constitutes a highly specific social situation and might itself be a factor with negative repercussions, e.g., on self-stigma. In other settings, the relationship between the investigated parameters with quality of life might take on a different form and dynamic owing to the breadth of the concept and its interconnectedness with various psychological, social and environmental factors.

Physical quality of life has emerged in our data as one of the major predictors of illness perception across groups, which proved particularly statistically robust in the F3* group. This suggests a need to address physical well-being in individuals suffering from severe mental illness. Physical activity has been shown to be one promising approach, with a systematic review showing positive effects of physical activity on quality of life across various mental and physical illnesses (Marquez et al., 2020). Exercise was also shown to have a favorable effect on various psychological outcome measures in patients suffering from substance use disorders (Giménez-Meseguer et al., 2020). The inclusion of exercise and physical activation in treatment regimens appears to be a viable approach targeting physical well-being and overall improvement in mental illnesses.

### 4.2 Illness perception and stages of change

Correlations in both groups between the *Coherence* and the *Treatment control* dimensions of the BIPQ, which reflect the subjective degree of understanding of one’s illness and the belief that treatment will help improve it, and the *Action* subscore of the FEVER suggest higher treatment motivation due to a better understanding of one’s illness. According to Prochaska’s Transtheoretical Model, progressing through the stages of change and reaching the *Action* stage is considered beneficial. Thus, a better understanding of the causes, course, and available treatment options appears to be advantageous and should be addressed in treatment settings, highlighting the importance of psychoeducation. A study on patients with bipolar disorder supports this assumption, showing that higher *Coherence*, along with *Treatment control*, was associated with greater medication adherence (Averous et al., 2018). Furthermore, a study on the effects of group psychoeducation and its mediators among patients with bipolar disorder revealed that illness perception was the primary mediator of the positive effects of psychoeducation on social functioning (Etain et al., 2018). Beyond the *Coherence* dimension, the *Action* subscore was also positively correlated with *Treatment control* in both groups, i.e., with the belief that the administered treatment will help the patient improve. Addressing *Treatment control* explicitly, e.g., by including information on treatment method efficacy and mechanisms in psychoeducation programs, may foster a more positive outlook on prognosis and willingness to change.

Notably, the associations of the stage of *Action* with illness perception dimensions showed a similar, yet not identical, pattern to that observed in a previous study of individuals with anorexia nervosa (Neu et al., 2025). Specifically, across all diagnostic entities, *Treatment Control* and *Coherence* were positively correlated with *Action*. However, *Emotional Representation* was negatively correlated with *Action* in the anorexia nervosa sample, whereas it was positively correlated in our sample of patients with affective disorders. Therefore, illness understanding and beliefs about the efficacy of offered treatment might be advantageous for progressing along the stages of change across different diagnostic entities, while emotional representation (the degree to which an individual sees themselves as emotionally affected by their disorder) might have a differential effect. Further investigation is needed to corroborate and clarify these discrepancies.

### 4.3 Limitations

The present investigation has several limitations. For example, due to the clinical nature of data collection, data was missing for several participants across the different scales. Additionally, the analyses employed with the lack of longitudinal data do not allow for statements on the causality and directionality of the reported associations. For example, the association between illness understanding and readiness to change may also reflect a greater willingness to learn about one’s illness among individuals who actively seek to improve their condition. Given the structure of available questionnaire (Hasler et al., 2003), we did not account for the full range of stages of the Transtheoretical Model of Behavior Change, which may be considered a shortcoming of our investigation. For a more comprehensive reflection of symptom load in the investigated patient groups, including measures of hypomanic and manic symptoms in the F3* group and psychotic symptoms in the F2* group would have complemented the findings sensibly. Contrasting the findings with the type and dosage of medications used and with the participants’ ethnicity would have elucidated these potentially relevant factors. Furthermore, we did not account for the presence of potential psychiatric comorbidities, which may have influenced the assessed parameters. Also, several variables with particular importance in the context of quality of life have not been considered, including stigma and self-stigma, childhood adversity, trauma, and attachment-related developmental factors. Future research would benefit from including said factors in investigations.

Participants were grouped according to ICD-10 diagnoses, as this is embedded in the data structure of our institution’s services. Particularly given the focus on subjective experience, our work could have profited from moving beyond a disorder-oriented classification and instead employ a more functional framework, e.g., the International Classification of Functioning, Disability and Health (ICF). ICF data are not part of our institution’s standard procedures and were not specifically collected for the current investigation, rendering a broader analysis of associations with factors such as patient resources and impairments, environmental barriers and facilitators, and participation restrictions impossible.

Our findings might lack generalizability and external validity owing to the specific setting in which data were collected. It cannot be assumed that all individuals suffering from a specific mental health disorder will be reached by psychiatric care institutions, and if so, be correctly diagnosed. Also, patients might object to undergoing or not be able to undergo in-patient treatment for various reasons. Similarly, patients might not be willing or able to participate in a questionnaire study. Also, in-patient treatment, particularly on units with a focus on short-term treatment of acute crises, represents a very specific setting in the overall treatment landscape. Our findings might have been influenced by the specificities of the context of data collection. On a statistical level, the employed complete-case analyses may introduce selection bias if data are not missing completely at random and that the reduced samples, particularly for some WHOQOL-BREF and BDI-II/BAI models, reduce precision and statistical power. In a supplementary sensitivity analysis comparing completers and non-completers of specific measures we identified several small-to-substantial baseline imbalances, e.g., regarding the age of participants. This may have introduced some bias. Therefore, our sample should be considered a specific subgroup of individuals suffering from the respective mental health disorders in a highly specific setting and their inclusion into the presented investigation might have been subject to a range of selection or collider biases, due to which our findings might not be readily generalizable to a broader context.

### 4.4 Between-group comparison, summary and clinical implications

We found substantial overlaps, but also some differences in the correlation between the examined factors between groups, e.g., in illness perceptions dimensions being correlated with anxiety measures only in the F3*. This disparity may be explained by the smaller number of participants in the F2* group leading to insufficient statistical power to support the same findings in both groups but may also reflect real between-group differences. The findings of the regression analyses point towards the same factors, namely *Physical Quality of Life* and depression scores, having the largest impact on illness perception dimensions in both groups, albeit on partially different dimensions and with greater statistical robustness in the affective sample. In Conclusion, our findings showed a great degree of overlap in the relationships between the investigated measures in both groups. The observed overlap may be consistent with a possible transdiagnostic pattern; however, this hypothesis requires confirmation through adequately powered studies with formal between-group comparisons.

In summary, our findings highlight the importance of illness perception in mental health disorders. It is associated with several clinical phenomena in individuals with psychotic and affective disorders. Thus, illness perception may be considered a significant aspect of individuals’ subjective experience across different mental health disorders that is highly relevant beyond the core symptoms of their respective disorders. Specifically, our findings highlight the importance of understanding of the illness and its treatment, as this may contribute to a greater readiness to change and seek treatment. Furthermore, physical quality of life and depression were strong predictors of several dimensions of illness perception.

In more concrete terms, our data point towards the importance of psychoeducation about the disorder and its treatment, which has been empirically established as an advantageous addition to treatment regimens (Alhadidi et al., 2020; Levrat et al., 2023; Carneiro et al., 2025), in the context of illness perception and readiness to change. They further suggest the need to specifically address physical well-being when treating mental health disorders, for example through the incorporation of physical activity interventions (Marquez et al., 2020), and also highlight the importance of considering depressive psychopathology in diagnostic and treatment processes, not only for those with affective disorders, but also with psychotic disorders.

Our findings add to the literature pointing towards the importance of psychological dimensions in severe mental illness, suggesting a necessity of employing therapeutic approaches beyond pharmacotherapy. Reflecting this and for the example of psychotic disorders, psychotherapy, while still rarely integrated in treatment settings, is increasingly recognized as an essential part of an adequate treatment regimen (Petzold, 2026). Relapse reduction, improved social integration, and the reduction of comorbid symptoms have been named as feasible treatment goals from the therapists’ perspective (Nübling et al., 2022). A recently published review further supports the importance of psychotherapy in psychotic illness, showing improvements after undergoing metacognitive therapies in first-episode psychosis with strongest evidence for improvements in the domain of cognitive insight (Requejo et al., 2026), which is arguably related to illness perception dimensions (Averous et al., 2020). Further widening the scope, considering our findings from a recovery- and participation-oriented perspective may be particularly relevant, as illness perceptions are embedded in treatment context, patient choice, and opportunities for social participation (Gühne et al., 2025; Standal et al., 2026).

## Data Availability

The data analyzed in this study is subject to the following licenses/restrictions: the datasets used in the presented article contain participants’ personalized information. Requests to access these datasets should be directed to Kilian Lommer, kilian.lommer@i-med.ac.at.
