# Supplementary material for: Comparison of factors of the subjective psychological condition of hospitalized individuals with schizophrenia spectrum and other psychotic disorders, or affective disorders: associations between illness perception, quality of life, affective and anxiety symptom severity, and readiness to change
Source: Front Psychol. 2026 Oct 2;17:1829834. doi: 10.3389/fpsyg.2026.1829834 (PMC13672533; doi:10.3389/fpsyg.2026.1829834)
Supplement: Supplementary file 1 [file Data_Sheet_1.PDF]

Supplementary Materials 1

Table of contents

Table S1: Variable-level availability and missingness by diagnostic group 2
Table S2. Model-specific analytic sample sizes for the primary regression models 3
Table S3. Patterns of questionnaire-block completeness by diagnostic group 5
Table S4. Baseline characteristics of completers and non-completers by diagnostic group and predictor block 5
Table S5. Multiple linear regression models of BIPQ domains on FEVER subscale scores in ICD-10 F2* patients 8
Table S6. Multiple linear regression models of BIPQ domains on WHOQOL-BREF domain scores in ICD-10 F2* patients 9
Table S7. Multiple linear regression models of BIPQ domains on BDI-II and BAI total scores in ICD-10 F2* patients 11
Table S8. Multiple linear regression models of BIPQ domains on FEVER subscale scores in ICD-10 F3* patients 12
Table S9. Multiple linear regression models of BIPQ domains on WHOQOL-BREF domain scores in ICD-10 F3* patients 13
Table S10. Multiple linear regression models of BIPQ domains on BDI-II and BAI total scores in ICD-10 F3* patients 15
Table S11. Sensitivity analysis: multiple linear regression models including all nine independent variables 15

Table S1: Variable-level availability and missingness by diagnostic group

Group	Variable	Total N	Available n	Missing n	Missing %
F2	Consequences	110	107	3	2.7
F2	Timeline	110	102	8	7.3
F2	Personal Control	110	106	4	3.6
F2	Treatment Control	110	105	5	4.5
F2	Identity	110	106	4	3.6
F2	Illness Concern	110	107	3	2.7
F2	Coherence	110	107	3	2.7
F2	Emotional Representation	110	106	4	3.6
F2	FEVER Precontemplation	110	89	21	19.1
F2	FEVER Contemplation	110	94	16	14.5
F2	FEVER Action	110	95	15	13.6
F2	WHOQOL-BREF Physical	110	50	60	54.5
F2	WHOQOL-BREF Psychological	110	50	60	54.5
F2	WHOQOL-BREF Social	110	50	60	54.5
F2	WHOQOL-BREF Environment	110	50	60	54.5
F2	BDI-II Total	110	50	60	54.5
F2	BAI Total	110	34	76	69.1
F2	A_BIPQ_total	110	100	10	9.1
F3	Consequences	261	250	11	4.2
F3	Timeline	261	240	21	8.0
F3	Personal Control	261	246	15	5.7
F3	Treatment Control	261	248	13	5.0
F3	Identity	261	249	12	4.6
F3	Illness Concern	261	250	11	4.2
F3	Coherence	261	250	11	4.2
F3	Emotional Representation	261	249	12	4.6
F3	FEVER Precontemplation	261	215	46	17.6
F3	FEVER Contemplation	261	225	36	13.8
F3	FEVER Action	261	229	32	12.3
F3	WHOQOL-BREF Physical	261	87	174	66.7
F3	WHOQOL-BREF Psychological	261	87	174	66.7

Group	Variable	Total N	Available n	Missing n	Missing %
F3	WHOQOL-BREF Social	261	86	175	67.0
F3	WHOQOL-BREF Environment	261	87	174	66.7
F3	BDI-II Total	261	88	173	66.3
F3	BAI Total	261	51	210	80.5
F3	A_BIPQ_total	261	233	28	10.7

Note. Total N denotes all participants in the respective diagnostic group; Available n denotes participants with a non-missing value for the variable; Missing n and Missing % denote the number and percentage of participants with a missing value. Percentages use Total N within the diagnostic group as the denominator.

Table S2. Model-specific analytic sample sizes for the primary regression models

Group	Outcome	Predictor block	Eligible N	Complete-case N	Excluded n	Excluded %
F2	Consequences	FEVER	110	84	26	23.6
F2	Timeline	FEVER	110	84	26	23.6
F2	Personal Control	FEVER	110	86	24	21.8
F2	Treatment Control	FEVER	110	85	25	22.7
F2	Identity	FEVER	110	86	24	21.8
F2	Illness Concern	FEVER	110	86	24	21.8
F2	Coherence	FEVER	110	86	24	21.8
F2	Emotional Representation	FEVER	110	85	25	22.7
F2	Consequences	WHOQOL_BREF	110	48	62	56.4
F2	Timeline	WHOQOL_BREF	110	46	64	58.2
F2	Personal Control	WHOQOL_BREF	110	48	62	56.4
F2	Treatment Control	WHOQOL_BREF	110	48	62	56.4
F2	Identity	WHOQOL_BREF	110	48	62	56.4
F2	Illness Concern	WHOQOL_BREF	110	49	61	55.5
F2	Coherence	WHOQOL_BREF	110	49	61	55.5
F2	Emotional Representation	WHOQOL_BREF	110	47	63	57.3
F2	Consequences	BDI_BAI	110	33	77	70.0
F2	Timeline	BDI_BAI	110	32	78	70.9
F2	Personal Control	BDI_BAI	110	33	77	70.0
F2	Treatment Control	BDI_BAI	110	33	77	70.0
F2	Identity	BDI_BAI	110	33	77	70.0

Group	Outcome	Predictor block	Eligible N	Complete-case N	Excluded n	Excluded %
F2	Illness Concern	BDI_BAI	110	33	77	70.0
F2	Coherence	BDI_BAI	110	33	77	70.0
F2	Emotional Representation	BDI_BAI	110	32	78	70.9
F3	Consequences	FEVER	261	204	57	21.8
F3	Timeline	FEVER	261	196	65	24.9
F3	Personal Control	FEVER	261	202	59	22.6
F3	Treatment Control	FEVER	261	202	59	22.6
F3	Identity	FEVER	261	203	58	22.2
F3	Illness Concern	FEVER	261	204	57	21.8
F3	Coherence	FEVER	261	204	57	21.8
F3	Emotional Representation	FEVER	261	204	57	21.8
F3	Consequences	WHOQOL_BREF	261	83	178	68.2
F3	Timeline	WHOQOL_BREF	261	78	183	70.1
F3	Personal Control	WHOQOL_BREF	261	82	179	68.6
F3	Treatment Control	WHOQOL_BREF	261	83	178	68.2
F3	Identity	WHOQOL_BREF	261	83	178	68.2
F3	Illness Concern	WHOQOL_BREF	261	82	179	68.6
F3	Coherence	WHOQOL_BREF	261	83	178	68.2
F3	Emotional Representation	WHOQOL_BREF	261	83	178	68.2
F3	Consequences	BDI_BAI	261	49	212	81.2
F3	Timeline	BDI_BAI	261	47	214	82.0
F3	Personal Control	BDI_BAI	261	49	212	81.2
F3	Treatment Control	BDI_BAI	261	49	212	81.2
F3	Identity	BDI_BAI	261	49	212	81.2
F3	Illness Concern	BDI_BAI	261	49	212	81.2
F3	Coherence	BDI_BAI	261	49	212	81.2
F3	Emotional Representation	BDI_BAI	261	49	212	81.2

Note. Eligible N denotes all participants in the respective diagnostic group. Complete-case N denotes participants with non-missing data for the specific BIPQ outcome and every predictor included in that model. Excluded n is the difference between Eligible N and Complete-case N; Excluded % uses Eligible N as the denominator. Complete-case status was therefore defined separately for each regression model.

Table S3. Patterns of questionnaire-block completeness by diagnostic group

Group	BIPQ	FEVER	WHOQOL-BREF	BDI-II/BAI	Pattern n	Pattern %
F2	Complete	Complete	Incomplete	Incomplete	45	40.9
F2	Complete	Complete	Complete	Complete	28	25.5
F2	Complete	Incomplete	Incomplete	Incomplete	11	10.0
F2	Complete	Complete	Complete	Incomplete	9	8.2
F2	Complete	Incomplete	Complete	Incomplete	4	3.6
F2	Complete	Incomplete	Complete	Complete	3	2.7
F2	Incomplete	Complete	Complete	Complete	3	2.7
F2	Incomplete	Incomplete	Incomplete	Incomplete	3	2.7
F2	Incomplete	Incomplete	Complete	Incomplete	2	1.8
F2	Incomplete	Complete	Complete	Incomplete	1	0.9
F2	Incomplete	Complete	Incomplete	Incomplete	1	0.9
F3	Complete	Complete	Incomplete	Incomplete	128	49.0
F3	Complete	Complete	Complete	Complete	38	14.6
F3	Complete	Incomplete	Incomplete	Incomplete	28	10.7
F3	Complete	Complete	Complete	Incomplete	27	10.3
F3	Incomplete	Complete	Incomplete	Incomplete	10	3.8
F3	Complete	Incomplete	Complete	Complete	9	3.4
F3	Incomplete	Incomplete	Incomplete	Incomplete	9	3.4
F3	Complete	Incomplete	Complete	Incomplete	3	1.1
F3	Incomplete	Incomplete	Complete	Incomplete	3	1.1
F3	Incomplete	Complete	Complete	Complete	2	0.8
F3	Incomplete	Complete	Complete	Incomplete	2	0.8
F3	Incomplete	Incomplete	Complete	Complete	2	0.8

Note. A questionnaire block was classified as complete only when all variables belonging to that block were observed. BIPQ completeness required all eight BIPQ domains; FEVER completeness required all three FEVER subscales; WHOQOL-BREF completeness required all four WHOQOL-BREF domains; and BDI-II/BAI completeness required both total scores. Pattern % is calculated within each diagnostic group. These descriptive block-level patterns do not determine inclusion in individual regression models, which used model-specific complete cases.

Table S4. Baseline characteristics of completers and non-completers by diagnostic group and predictor block

Group	Predictor block	Characteristic	Non-completer	Completer	SMD
F2	FEVER	n	28	82	
F2	FEVER	Age, mean (SD)	44.89 (13.56)	42.46 (14.60)	0.172

Group	Predictor block	Characteristic	Non-completer	Completer	SMD
F2	FEVER	Sex			0.213
F2	FEVER	Female	11 (39.3)	29 (35.4)	
F2	FEVER	Male	16 (57.1)	46 (56.1)	
F2	FEVER	NA	1 (3.6)	7 (8.5)	
F2	FEVER	Mother tongue			0.182
F2	FEVER	German	16 (57.1)	54 (65.9)	
F2	FEVER	Other language	5 (17.9)	11 (13.4)	
F2	FEVER	NA	7 (25.0)	17 (20.7)	
F2	FEVER	Nationality			0.280
F2	FEVER	Austria	15 (53.6)	55 (67.1)	
F2	FEVER	Other nationality	5 (17.9)	11 (13.4)	
F2	FEVER	NA	8 (28.6)	16 (19.5)	
F2	WHOQOL_BREF	n	66	44	
F2	WHOQOL_BREF	Age, mean (SD)	44.73 (14.09)	40.61 (14.47)	0.288
F2	WHOQOL_BREF	Sex			0.273
F2	WHOQOL_BREF	Female	21 (31.8)	19 (43.2)	
F2	WHOQOL_BREF	Male	39 (59.1)	23 (52.3)	
F2	WHOQOL_BREF	NA	6 (9.1)	2 (4.5)	
F2	WHOQOL_BREF	Mother tongue			0.453
F2	WHOQOL_BREF	German	39 (59.1)	31 (70.5)	
F2	WHOQOL_BREF	Other language	8 (12.1)	8 (18.2)	
F2	WHOQOL_BREF	NA	19 (28.8)	5 (11.4)	
F2	WHOQOL_BREF	Nationality			0.402
F2	WHOQOL_BREF	Austria	41 (62.1)	29 (65.9)	
F2	WHOQOL_BREF	Other nationality	7 (10.6)	9 (20.5)	
F2	WHOQOL_BREF	NA	18 (27.3)	6 (13.6)	
F2	BDI_BAI	n	79	31	
F2	BDI_BAI	Age, mean (SD)	44.82 (13.97)	38.65 (14.45)	0.435
F2	BDI_BAI	Sex			0.075
F2	BDI_BAI	Female	28 (35.4)	12 (38.7)	
F2	BDI_BAI	Male	45 (57.0)	17 (54.8)	
F2	BDI_BAI	NA	6 (7.6)	2 (6.5)	

Group	Predictor block	Characteristic	Non-completer	Completer	SMD
F2	BDI_BAI	Mother tongue			0.320
F2	BDI_BAI	German	48 (60.8)	22 (71.0)	
F2	BDI_BAI	Other language	11 (13.9)	5 (16.1)	
F2	BDI_BAI	NA	20 (25.3)	4 (12.9)	
F2	BDI_BAI	Nationality			0.342
F2	BDI_BAI	Austria	49 (62.0)	21 (67.7)	
F2	BDI_BAI	Other nationality	10 (12.7)	6 (19.4)	
F2	BDI_BAI	NA	20 (25.3)	4 (12.9)	
F3	FEVER	n	68	193	
F3	FEVER	Age, mean (SD)	56.09 (16.78)	44.40 (15.83)	0.716
F3	FEVER	Sex			0.389
F3	FEVER	Female	35 (51.5)	66 (34.2)	
F3	FEVER	Male	26 (38.2)	110 (57.0)	
F3	FEVER	NA	7 (10.3)	17 (8.8)	
F3	FEVER	Mother tongue			0.290
F3	FEVER	German	34 (50.0)	123 (63.7)	
F3	FEVER	Other language	11 (16.2)	19 (9.8)	
F3	FEVER	NA	23 (33.8)	51 (26.4)	
F3	FEVER	Nationality			0.237
F3	FEVER	Austria	38 (55.9)	116 (60.1)	
F3	FEVER	Other nationality	6 (8.8)	27 (14.0)	
F3	FEVER	NA	24 (35.3)	50 (25.9)	
F3	WHOQOL_BREF	n	184	77	
F3	WHOQOL_BREF	Age, mean (SD)	47.91 (17.42)	46.35 (15.47)	0.095
F3	WHOQOL_BREF	Sex			0.234
F3	WHOQOL_BREF	Female	65 (35.3)	36 (46.8)	
F3	WHOQOL_BREF	Male	101 (54.9)	35 (45.5)	
F3	WHOQOL_BREF	NA	18 (9.8)	6 (7.8)	
F3	WHOQOL_BREF	Mother tongue			0.217
F3	WHOQOL_BREF	German	108 (58.7)	49 (63.6)	
F3	WHOQOL_BREF	Other language	19 (10.3)	11 (14.3)	
F3	WHOQOL_BREF	NA	57 (31.0)	17 (22.1)	

Group	Predictor block	Characteristic	Non-completer	Completer	SMD
F3	WHOQOL_BREF	Nationality			0.311
F3	WHOQOL_BREF	Austria	105 (57.1)	49 (63.6)	
F3	WHOQOL_BREF	Other nationality	20 (10.9)	13 (16.9)	
F3	WHOQOL_BREF	NA	59 (32.1)	15 (19.5)	
F3	BDI_BAI	n	214	47	
F3	BDI_BAI	Age, mean (SD)	47.54 (17.33)	47.02 (14.65)	0.032
F3	BDI_BAI	Sex			0.279
F3	BDI_BAI	Female	79 (36.9)	22 (46.8)	
F3	BDI_BAI	Male	113 (52.8)	23 (48.9)	
F3	BDI_BAI	NA	22 (10.3)	2 (4.3)	
F3	BDI_BAI	Mother tongue			0.213
F3	BDI_BAI	German	125 (58.4)	32 (68.1)	
F3	BDI_BAI	Other language	25 (11.7)	5 (10.6)	
F3	BDI_BAI	NA	64 (29.9)	10 (21.3)	
F3	BDI_BAI	Nationality			0.361
F3	BDI_BAI	Austria	120 (56.1)	34 (72.3)	
F3	BDI_BAI	Other nationality	28 (13.1)	5 (10.6)	
F3	BDI_BAI	NA	66 (30.8)	8 (17.0)	

Note. For this sensitivity analysis, a completer had complete data for all eight BIPQ outcomes and all predictors in the corresponding predictor block; all other participants were classified as non-completers. Age is summarized as mean (SD), and categorical variables as n (%). SMD = standardized mean difference. SMDs are reported descriptively to assess potential baseline imbalance: values close to 0 indicate similar baseline distributions between completers and non-completers. As a descriptive rule of thumb, absolute SMDs around 0.10, 0.20, and 0.50 can be viewed as small, modest, and substantial imbalance, respectively; the pattern and clinical relevance should be interpreted rather than treating these as formal significance thresholds; no additional hypothesis tests were performed. Sex was coded as female or male; mother tongue as German or other language; and citizenship as Austria or other nationality. Missing demographic values remained missing.

Table S5. Multiple linear regression models of BIPQ domains on FEVER subscale scores in ICD-10 F2* patients

Outcome	N	Predictor	B (SE)	95% CI for B	β	95% CI for β	p	p FDR	VIF	R²	Adjusted R²
Consequences	84	FEVER Precontemplation	1.11 (0.40)	0.32 to 1.90	0.29	0.08 to 0.49	0.006	0.081	1.05	0.20	0.17
	84	FEVER Contemplation	1.92 (0.69)	0.55 to 3.30	0.43	0.12 to 0.73	0.007	0.081	2.35	0.20	0.17
	84	FEVER Action	-1.48 (0.72)	-2.92 to -0.04	-0.31	-0.62 to -0.01	0.044	0.210	2.33	0.20	0.17
Timeline	84	FEVER Precontemplation	0.85 (0.47)	-0.09 to 1.79	0.20	-0.02 to 0.42	0.074	0.255	1.05	0.05	0.01
	84	FEVER Contemplation	0.19 (0.82)	-1.44 to 1.83	0.04	-0.30 to 0.38	0.814	0.849	2.41	0.05	0.01

Outcome	N	Predictor	B (SE)	95% CI for B	β	95% CI for β	p	p FDR	VIF	R²	Adjusted R²
	84	FEVER Action	0.21 (0.86)	-1.50 to 1.92	0.04	-0.29 to 0.38	0.808	0.849	2.37	0.05	0.01
Personal Control	86	FEVER Precontemplation	-0.15 (0.40)	-0.95 to 0.65	-0.04	-0.26 to 0.18	0.711	0.849	1.05	0.02	-0.02
	86	FEVER Contemplation	-0.65 (0.71)	-2.06 to 0.76	-0.16	-0.49 to 0.18	0.363	0.592	2.41	0.02	-0.02
	86	FEVER Action	0.85 (0.74)	-0.62 to 2.32	0.19	-0.14 to 0.53	0.252	0.504	2.37	0.02	-0.02
Treatment Control	85	FEVER Precontemplation	0.39 (0.36)	-0.33 to 1.11	0.12	-0.10 to 0.34	0.280	0.517	1.05	0.04	0.01
	85	FEVER Contemplation	-0.15 (0.63)	-1.40 to 1.10	-0.04	-0.37 to 0.30	0.814	0.849	2.40	0.04	0.01
	85	FEVER Action	0.81 (0.66)	-0.50 to 2.12	0.21	-0.13 to 0.54	0.221	0.481	2.37	0.04	0.01
Identity	86	FEVER Precontemplation	0.53 (0.43)	-0.32 to 1.38	0.14	-0.08 to 0.36	0.220	0.481	1.05	0.04	0.00
	86	FEVER Contemplation	0.68 (0.75)	-0.82 to 2.18	0.15	-0.18 to 0.49	0.370	0.592	2.41	0.04	0.00
	86	FEVER Action	-0.22 (0.78)	-1.78 to 1.34	-0.05	-0.38 to 0.28	0.779	0.849	2.37	0.04	0.00
Illness Concern	86	FEVER Precontemplation	0.85 (0.45)	-0.05 to 1.75	0.21	-0.01 to 0.42	0.063	0.254	1.05	0.07	0.03
	86	FEVER Contemplation	0.61 (0.79)	-0.97 to 2.19	0.13	-0.20 to 0.46	0.443	0.645	2.41	0.07	0.03
	86	FEVER Action	0.07 (0.82)	-1.57 to 1.71	0.01	-0.31 to 0.34	0.935	0.935	2.37	0.07	0.03
Coherence	86	FEVER Precontemplation	-0.31 (0.41)	-1.13 to 0.51	-0.08	-0.30 to 0.13	0.457	0.645	1.05	0.08	0.05
	86	FEVER Contemplation	-1.10 (0.73)	-2.54 to 0.35	-0.25	-0.57 to 0.08	0.135	0.405	2.41	0.08	0.05
	86	FEVER Action	1.85 (0.75)	0.35 to 3.35	0.40	0.08 to 0.72	0.016	0.117	2.37	0.08	0.05
Emotional Representation	85	FEVER Precontemplation	0.52 (0.40)	-0.26 to 1.31	0.14	-0.07 to 0.35	0.190	0.481	1.05	0.14	0.11
	85	FEVER Contemplation	1.66 (0.69)	0.27 to 3.04	0.38	0.06 to 0.70	0.019	0.117	2.39	0.14	0.11
	85	FEVER Action	-0.37 (0.72)	-1.80 to 1.07	-0.08	-0.40 to 0.23	0.612	0.816	2.36	0.14	0.11

Note. *B = unstandardized regression coefficient; SE = standard error; CI = confidence interval; β = standardized regression coefficient; VIF = variance inflation factor; R² = coefficient of determination. The 95% CIs are reported separately for B and β. p values are two-sided nominal p values; p FDR values were adjusted using the Benjamini-Hochberg false discovery rate procedure within each diagnostic group and predictor block. Each regression used model-specific complete cases.*

Table S6. Multiple linear regression models of BIPQ domains on WHOQOL-BREF domain scores in ICD-10 F2* patients

Outcome	N	Predictor	B (SE)	95% CI for B	β	95% CI for β	p	p FDR	VIF	R²	Adjusted R²

Outcome	N	Predictor	B (SE)	95% CI for B	β	95% CI for β	p	p FDR	VIF	R^2	Adjusted R^2
Consequences	48	WHOQOL-BREF Physical	-0.08 (0.03)	-0.14 to -0.02	-0.42	-0.75 to -0.08	0.015	0.081	2.11	0.44	0.39
	48	WHOQOL-BREF Psychological	-0.07 (0.02)	-0.11 to -0.02	-0.43	-0.73 to -0.13	0.006	0.053	1.73	0.44	0.39
	48	WHOQOL-BREF Social	0.01 (0.02)	-0.02 to 0.05	0.10	-0.17 to 0.37	0.462	0.692	1.39	0.44	0.39
	48	WHOQOL-BREF Environment	0.02 (0.02)	-0.03 to 0.07	0.12	-0.21 to 0.45	0.464	0.692	2.04	0.44	0.39
Timeline	46	WHOQOL-BREF Physical	-0.11 (0.04)	-0.19 to -0.02	-0.53	-0.95 to -0.12	0.012	0.078	2.32	0.27	0.20
	46	WHOQOL-BREF Psychological	-0.02 (0.03)	-0.08 to 0.04	-0.13	-0.48 to 0.22	0.461	0.692	1.70	0.27	0.20
	46	WHOQOL-BREF Social	0.01 (0.02)	-0.04 to 0.05	0.05	-0.27 to 0.36	0.773	0.854	1.37	0.27	0.20
	46	WHOQOL-BREF Environment	0.02 (0.03)	-0.03 to 0.08	0.16	-0.23 to 0.54	0.413	0.692	2.04	0.27	0.20
Personal Control	48	WHOQOL-BREF Physical	0.02 (0.03)	-0.04 to 0.09	0.16	-0.27 to 0.58	0.455	0.692	2.11	0.10	0.02
	48	WHOQOL-BREF Psychological	0.03 (0.02)	-0.01 to 0.08	0.26	-0.11 to 0.64	0.166	0.383	1.66	0.10	0.02
	48	WHOQOL-BREF Social	0.01 (0.02)	-0.03 to 0.05	0.11	-0.23 to 0.45	0.531	0.708	1.36	0.10	0.02
	48	WHOQOL-BREF Environment	-0.03 (0.02)	-0.08 to 0.02	-0.27	-0.68 to 0.14	0.186	0.396	1.98	0.10	0.02
Treatment Control	48	WHOQOL-BREF Physical	0.01 (0.03)	-0.06 to 0.08	0.05	-0.39 to 0.48	0.833	0.889	2.11	0.04	-0.05
	48	WHOQOL-BREF Psychological	0.02 (0.02)	-0.03 to 0.07	0.13	-0.26 to 0.52	0.498	0.692	1.66	0.04	-0.05
	48	WHOQOL-BREF Social	0.02 (0.02)	-0.02 to 0.06	0.15	-0.20 to 0.50	0.385	0.692	1.36	0.04	-0.05
	48	WHOQOL-BREF Environment	-0.01 (0.03)	-0.06 to 0.04	-0.11	-0.53 to 0.32	0.616	0.736	1.98	0.04	-0.05
Identity	48	WHOQOL-BREF Physical	-0.06 (0.03)	-0.12 to 0.01	-0.33	-0.70 to 0.04	0.076	0.241	2.11	0.32	0.25
	48	WHOQOL-BREF Psychological	-0.04 (0.02)	-0.08 to 0.01	-0.27	-0.60 to 0.05	0.100	0.266	1.66	0.32	0.25
	48	WHOQOL-BREF Social	0.04 (0.02)	0.00 to 0.07	0.31	0.01 to 0.61	0.040	0.184	1.36	0.32	0.25
	48	WHOQOL-BREF Environment	-0.01 (0.02)	-0.06 to 0.03	-0.09	-0.45 to 0.26	0.603	0.736	1.98	0.32	0.25
Illness Concern	49	WHOQOL-BREF Physical	-0.09 (0.03)	-0.16 to -0.03	-0.49	-0.82 to -0.15	0.006	0.053	2.14	0.43	0.38
	49	WHOQOL-BREF Psychological	-0.07 (0.02)	-0.11 to -0.02	-0.43	-0.73 to -0.12	0.007	0.053	1.71	0.43	0.38
	49	WHOQOL-BREF Social	-0.00 (0.02)	-0.04 to 0.04	-0.01	-0.28 to 0.27	0.968	0.977	1.41	0.43	0.38
	49	WHOQOL-BREF	0.05 (0.02)	0.00 to	0.34	0.01 to	0.047	0.187	2.08	0.43	0.38

Outcome	N	Predictor	B (SE)	95% CI for B	β	95% CI for β	p	p FDR	VIF	R^2	Adjusted R^2
		Environment		0.10		0.67					
Coherence	49	WHOQOL-BREF Physical	-0.00 (0.03)	-0.07 to 0.07	-0.01	-0.42 to 0.41	0.977	0.977	2.14	0.12	0.04
	49	WHOQOL-BREF Psychological	0.03 (0.02)	-0.02 to 0.08	0.26	-0.11 to 0.63	0.167	0.383	1.71	0.12	0.04
	49	WHOQOL-BREF Social	0.01 (0.02)	-0.03 to 0.05	0.08	-0.25 to 0.42	0.621	0.736	1.41	0.12	0.04
	49	WHOQOL-BREF Environment	0.01 (0.03)	-0.04 to 0.06	0.09	-0.32 to 0.50	0.661	0.756	2.08	0.12	0.04
Emotional Representation	47	WHOQOL-BREF Physical	-0.05 (0.03)	-0.11 to 0.01	-0.30	-0.64 to 0.04	0.078	0.241	2.19	0.47	0.42
	47	WHOQOL-BREF Psychological	-0.09 (0.02)	-0.13 to -0.05	-0.61	-0.90 to -0.32	<.001	0.004	1.66	0.47	0.42
	47	WHOQOL-BREF Social	0.01 (0.02)	-0.02 to 0.05	0.09	-0.17 to 0.35	0.476	0.692	1.31	0.47	0.42
	47	WHOQOL-BREF Environment	0.04 (0.02)	-0.01 to 0.08	0.28	-0.04 to 0.60	0.083	0.241	1.95	0.47	0.42

Note. B = unstandardized regression coefficient; SE = standard error; CI = confidence interval; β = standardized regression coefficient; VIF = variance inflation factor; R^2 = coefficient of determination. The 95% CIs are reported separately for B and β. p values are two-sided nominal p values; p FDR values were adjusted using the Benjamini-Hochberg false discovery rate procedure within each diagnostic group and predictor block. Each regression used model-specific complete cases.

Table S7. Multiple linear regression models of BIPQ domains on BDI-II and BAI total scores in ICD-10 F2* patients

Outcome	N	Predictor	B (SE)	95% CI for B	β	95% CI for β	p	p FDR	VIF	R^2	Adjusted R^2
Consequences	33	BDI-II Total	0.15 (0.05)	0.04 to 0.27	0.45	0.13 to 0.77	0.008	0.042	1.12	0.33	0.28
	33	BAI Total	0.05 (0.04)	-0.02 to 0.12	0.23	-0.09 to 0.56	0.148	0.474	1.12	0.33	0.28
Timeline	32	BDI-II Total	0.02 (0.07)	-0.12 to 0.17	0.07	-0.33 to 0.46	0.734	0.839	1.11	0.02	-0.04
	32	BAI Total	0.03 (0.05)	-0.07 to 0.12	0.12	-0.27 to 0.52	0.536	0.693	1.11	0.02	-0.04
Personal Control	33	BDI-II Total	-0.06 (0.05)	-0.17 to 0.05	-0.20	-0.58 to 0.18	0.285	0.652	1.12	0.08	0.02
	33	BAI Total	-0.03 (0.04)	-0.10 to 0.04	-0.14	-0.52 to 0.23	0.444	0.693	1.12	0.08	0.02
Treatment Control	33	BDI-II Total	-0.00 (0.05)	-0.11 to 0.11	-0.01	-0.40 to 0.39	0.978	0.978	1.12	0.01	-0.05
	33	BAI Total	0.02 (0.03)	-0.05 to 0.09	0.11	-0.28 to 0.50	0.563	0.693	1.12	0.01	-0.05
Identity	33	BDI-II Total	0.07 (0.06)	-0.05 to 0.19	0.22	-0.16 to 0.59	0.252	0.652	1.12	0.09	0.03
	33	BAI Total	0.03 (0.04)	-0.05 to 0.11	0.14	-0.23 to 0.52	0.438	0.693	1.12	0.09	0.03
Illness Concern	33	BDI-II Total	0.14 (0.06)	0.02 to 0.26	0.40	0.06 to 0.75	0.023	0.091	1.12	0.24	0.18
	33	BAI Total	0.04 (0.04)	-0.04 to 0.12	0.17	-0.18 to 0.51	0.328	0.656	1.12	0.24	0.18

Outcome	N	Predictor	B (SE)	95% CI for B	β	95% CI for β	p	p FDR	VIF	R²	Adjusted R²
Coherence	33	BDI-II Total	-0.16 (0.05)	-0.25 to -0.06	-0.55	-0.88 to -0.22	0.002	0.016	1.12	0.29	0.24
	33	BAI Total	0.01 (0.03)	-0.05 to 0.07	0.04	-0.29 to 0.37	0.819	0.873	1.12	0.29	0.24
Emotional Representation	32	BDI-II Total	0.23 (0.05)	0.14 to 0.33	0.70	0.41 to 0.99	<.001	<.001	1.09	0.46	0.42
	32	BAI Total	-0.02 (0.03)	-0.08 to 0.04	-0.09	-0.38 to 0.21	0.551	0.693	1.09	0.46	0.42

Note. B = unstandardized regression coefficient; SE = standard error; CI = confidence interval; β = standardized regression coefficient; VIF = variance inflation factor; R² = coefficient of determination. The 95% CIs are reported separately for B and β. p values are two-sided nominal p values; p FDR values were adjusted using the Benjamini-Hochberg false discovery rate procedure within each diagnostic group and predictor block. Each regression used model-specific complete cases.

Table S8. Multiple linear regression models of BIPQ domains on FEVER subscale scores in ICD-10 F3* patients

Outcome	N	Predictor	B (SE)	95% CI for B	β	95% CI for β	p	p FDR	VIF	R²	Adjusted R²
Consequences	204	FEVER Precontemplation	0.50 (0.17)	0.16 to 0.84	0.22	0.07 to 0.36	0.004	0.031	1.16	0.06	0.05
	204	FEVER Contemplation	0.42 (0.32)	-0.21 to 1.06	0.12	-0.06 to 0.31	0.188	0.410	1.89	0.06	0.05
	204	FEVER Action	-0.38 (0.23)	-0.82 to 0.07	-0.15	-0.33 to 0.03	0.097	0.289	1.74	0.06	0.05
Timeline	196	FEVER Precontemplation	0.23 (0.27)	-0.31 to 0.77	0.06	-0.09 to 0.22	0.404	0.623	1.14	0.01	-0.01
	196	FEVER Contemplation	0.08 (0.51)	-0.93 to 1.09	0.01	-0.18 to 0.21	0.882	0.993	1.88	0.01	-0.01
	196	FEVER Action	-0.36 (0.36)	-1.07 to 0.35	-0.10	-0.28 to 0.09	0.315	0.580	1.76	0.01	-0.01
Personal Control	202	FEVER Precontemplation	-0.14 (0.26)	-0.65 to 0.37	-0.04	-0.19 to 0.11	0.582	0.735	1.15	0.05	0.04
	202	FEVER Contemplation	-0.79 (0.49)	-1.75 to 0.17	-0.15	-0.34 to 0.03	0.108	0.289	1.86	0.05	0.04
	202	FEVER Action	1.12 (0.34)	0.45 to 1.79	0.30	0.12 to 0.48	0.001	0.013	1.72	0.05	0.04
Treatment Control	202	FEVER Precontemplation	0.10 (0.21)	-0.32 to 0.51	0.03	-0.10 to 0.17	0.645	0.774	1.15	0.19	0.18
	202	FEVER Contemplation	1.33 (0.39)	0.55 to 2.11	0.30	0.12 to 0.47	<.001	0.013	1.87	0.19	0.18
	202	FEVER Action	0.55 (0.28)	0.00 to 1.09	0.17	0.00 to 0.33	0.049	0.223	1.73	0.19	0.18
Identity	203	FEVER Precontemplation	0.02 (0.23)	-0.43 to 0.48	0.01	-0.14 to 0.16	0.925	0.993	1.16	0.02	0.00
	203	FEVER Contemplation	0.31 (0.43)	-0.54 to 1.16	0.07	-0.12 to 0.26	0.479	0.641	1.89	0.02	0.00
	203	FEVER Action	0.25 (0.30)	-0.35 to 0.84	0.08	-0.11 to 0.26	0.415	0.623	1.74	0.02	0.00

Outcome	N	Predictor	B (SE)	95% CI for B	β	95% CI for β	p	p FDR	VIF	R²	Adjusted R²
Illness Concern	204	FEVER Precontemplation	-0.00 (0.25)	-0.49 to 0.48	- 0.00	-0.15 to 0.15	0.993	0.993	1.16	0.02	0.00
	204	FEVER Contemplation	0.63 (0.46)	-0.28 to 1.54	0.13	-0.06 to 0.32	0.174	0.410	1.89	0.02	0.00
	204	FEVER Action	0.02 (0.32)	-0.62 to 0.66	0.01	-0.18 to 0.19	0.953	0.993	1.74	0.02	0.00
Coherence	204	FEVER Precontemplation	0.56 (0.29)	-0.01 to 1.14	0.14	-0.00 to 0.29	0.056	0.223	1.16	0.05	0.04
	204	FEVER Contemplation	-0.96 (0.55)	-2.04 to 0.12	- 0.17	-0.35 to 0.02	0.082	0.283	1.89	0.05	0.04
	204	FEVER Action	0.98 (0.39)	0.22 to 1.75	0.23	0.05 to 0.41	0.012	0.070	1.74	0.05	0.04
Emotional Representation	204	FEVER Precontemplation	0.14 (0.20)	-0.25 to 0.52	0.05	-0.09 to 0.20	0.480	0.641	1.16	0.04	0.02
	204	FEVER Contemplation	0.35 (0.37)	-0.37 to 1.07	0.09	-0.10 to 0.28	0.338	0.580	1.89	0.04	0.02
	204	FEVER Action	0.25 (0.26)	-0.26 to 0.75	0.09	-0.09 to 0.27	0.337	0.580	1.74	0.04	0.02

Note. B = unstandardized regression coefficient; SE = standard error; CI = confidence interval; β = standardized regression coefficient; VIF = variance inflation factor; R² = coefficient of determination. The 95% CIs are reported separately for B and β. p values are two-sided nominal p values; p FDR values were adjusted using the Benjamini-Hochberg false discovery rate procedure within each diagnostic group and predictor block. Each regression used model-specific complete cases.

Table S9. Multiple linear regression models of BIPQ domains on WHOQOL-BREF domain scores in ICD-10 F3* patients

Outcome	N	Predictor	B (SE)	95% CI for B	β	95% CI for β	p	p FDR	VIF	R²	Adjusted R²
Consequences	83	WHOQOL-BREF Physical	-0.05 (0.01)	-0.08 to -0.02	-0.47	-0.73 to -0.22	<.001	0.006	1.83	0.31	0.28
	83	WHOQOL-BREF Psychological	-0.02 (0.01)	-0.05 to -0.00	-0.25	-0.49 to -0.01	0.040	0.129	1.65	0.31	0.28
	83	WHOQOL-BREF Social	0.01 (0.01)	-0.01 to 0.03	0.10	-0.12 to 0.32	0.370	0.539	1.41	0.31	0.28
	83	WHOQOL-BREF Environment	0.02 (0.01)	-0.01 to 0.04	0.16	-0.07 to 0.39	0.176	0.339	1.50	0.31	0.28
Timeline	78	WHOQOL-BREF Physical	-0.06 (0.02)	-0.10 to -0.02	-0.40	-0.67 to -0.14	0.004	0.023	1.76	0.26	0.22
	78	WHOQOL-BREF Psychological	-0.01 (0.02)	-0.04 to 0.02	-0.08	-0.33 to 0.18	0.543	0.621	1.59	0.26	0.22
	78	WHOQOL-BREF Social	-0.02 (0.01)	-0.05 to 0.01	-0.17	-0.42 to 0.08	0.190	0.339	1.56	0.26	0.22
	78	WHOQOL-BREF Environment	0.01 (0.02)	-0.02 to 0.05	0.09	-0.15 to 0.34	0.443	0.617	1.49	0.26	0.22
Personal Control	82	WHOQOL-BREF Physical	0.03 (0.02)	-0.01 to 0.07	0.18	-0.09 to 0.46	0.186	0.339	1.83	0.21	0.17
	82	WHOQOL-BREF Psychological	0.06 (0.02)	0.02 to 0.09	0.40	0.14 to 0.66	0.003	0.022	1.65	0.21	0.17

Outcome	N	Predictor	B (SE)	95% CI for B	β	95% CI for β	p	p FDR	VIF	R²	Adjusted R²
	82	WHOQOL-BREF Social	-0.01 (0.01)	-0.04 to 0.02	-0.08	-0.31 to 0.16	0.534	0.621	1.41	0.21	0.17
	82	WHOQOL-BREF Environment	-0.04 (0.02)	-0.08 to -0.00	-0.27	-0.52 to -0.03	0.030	0.113	1.49	0.21	0.17
Treatment Control	83	WHOQOL-BREF Physical	-0.01 (0.02)	-0.04 to 0.03	-0.04	-0.33 to 0.25	0.773	0.800	1.83	0.11	0.07
	83	WHOQOL-BREF Psychological	0.04 (0.02)	0.01 to 0.08	0.36	0.08 to 0.63	0.011	0.051	1.65	0.11	0.07
	83	WHOQOL-BREF Social	-0.01 (0.01)	-0.04 to 0.01	-0.11	-0.37 to 0.14	0.370	0.539	1.42	0.11	0.07
	83	WHOQOL-BREF Environment	0.01 (0.02)	-0.02 to 0.05	0.09	-0.17 to 0.34	0.508	0.621	1.48	0.11	0.07
Identity	83	WHOQOL-BREF Physical	-0.09 (0.02)	-0.13 to -0.06	-0.66	-0.91 to -0.42	<.001	<.001	1.88	0.38	0.35
	83	WHOQOL-BREF Psychological	-0.01 (0.01)	-0.04 to 0.01	-0.12	-0.35 to 0.11	0.308	0.502	1.67	0.38	0.35
	83	WHOQOL-BREF Social	0.02 (0.01)	-0.00 to 0.04	0.18	-0.03 to 0.39	0.092	0.227	1.41	0.38	0.35
	83	WHOQOL-BREF Environment	0.02 (0.01)	-0.01 to 0.05	0.18	-0.04 to 0.39	0.106	0.242	1.49	0.38	0.35
Illness Concern	82	WHOQOL-BREF Physical	-0.06 (0.02)	-0.09 to -0.02	-0.44	-0.70 to -0.18	0.001	0.014	1.88	0.30	0.26
	82	WHOQOL-BREF Psychological	-0.03 (0.01)	-0.06 to -0.00	-0.27	-0.51 to -0.02	0.032	0.113	1.65	0.30	0.26
	82	WHOQOL-BREF Social	0.00 (0.01)	-0.02 to 0.03	0.03	-0.19 to 0.26	0.775	0.800	1.41	0.30	0.26
	82	WHOQOL-BREF Environment	0.03 (0.02)	-0.00 to 0.06	0.20	-0.03 to 0.44	0.088	0.227	1.51	0.30	0.26
Coherence	83	WHOQOL-BREF Physical	-0.02 (0.02)	-0.07 to 0.02	-0.15	-0.45 to 0.15	0.314	0.502	1.83	0.04	-0.00
	83	WHOQOL-BREF Psychological	0.03 (0.02)	-0.01 to 0.07	0.21	-0.08 to 0.49	0.150	0.319	1.65	0.04	-0.00
	83	WHOQOL-BREF Social	0.01 (0.01)	-0.02 to 0.03	0.05	-0.22 to 0.31	0.731	0.800	1.41	0.04	-0.00
	83	WHOQOL-BREF Environment	0.01 (0.02)	-0.03 to 0.05	0.09	-0.18 to 0.36	0.509	0.621	1.50	0.04	-0.00
Emotional Representation	83	WHOQOL-BREF Physical	-0.03 (0.01)	-0.06 to 0.00	-0.26	-0.52 to 0.01	0.059	0.171	1.88	0.26	0.22
	83	WHOQOL-BREF Psychological	-0.03 (0.01)	-0.06 to -0.01	-0.36	-0.61 to -0.11	0.006	0.030	1.67	0.26	0.22
	83	WHOQOL-BREF Social	0.00 (0.01)	-0.02 to 0.02	0.02	-0.21 to 0.25	0.893	0.893	1.41	0.26	0.22
	83	WHOQOL-BREF Environment	0.01 (0.01)	-0.02 to 0.03	0.08	-0.16 to 0.31	0.523	0.621	1.49	0.26	0.22

Note. B = unstandardized regression coefficient; SE = standard error; CI = confidence interval; β = standardized regression coefficient; VIF = variance inflation factor; R^2 = coefficient of determination. The 95% CIs are reported separately for B and β. p values are two-sided nominal p values; p FDR values were adjusted using the Benjamini-Hochberg false discovery rate procedure within each diagnostic group and predictor block. Each regression used model-specific complete cases.

Table S10. Multiple linear regression models of BIPQ domains on BDI-II and BAI total scores in ICD-10 F3* patients

Outcome	N	Predictor	B (SE)	95% CI for B	β	95% CI for β	p	p FDR	VIF	R²	Adjusted R²
Consequences	49	BDI-II Total	0.08 (0.02)	0.03 to 0.12	0.54	0.22 to 0.86	0.001	0.009	1.72	0.33	0.30
	49	BAI Total	0.01 (0.03)	-0.05 to 0.06	0.04	-0.28 to 0.36	0.800	0.842	1.72	0.33	0.30
Timeline	47	BDI-II Total	0.08 (0.03)	0.01 to 0.15	0.39	0.07 to 0.71	0.018	0.059	1.61	0.30	0.27
	47	BAI Total	0.05 (0.04)	-0.03 to 0.13	0.21	-0.11 to 0.53	0.195	0.311	1.61	0.30	0.27
Personal Control	49	BDI-II Total	-0.08 (0.04)	-0.15 to 0.00	-0.37	-0.73 to 0.00	0.053	0.141	1.72	0.10	0.06
	49	BAI Total	0.02 (0.04)	-0.06 to 0.11	0.10	-0.27 to 0.47	0.580	0.750	1.72	0.10	0.06
Treatment Control	49	BDI-II Total	-0.11 (0.03)	-0.18 to -0.05	-0.62	-0.96 to -0.28	<.001	0.009	1.72	0.25	0.21
	49	BAI Total	0.05 (0.04)	-0.02 to 0.13	0.26	-0.08 to 0.59	0.134	0.238	1.72	0.25	0.21
Identity	49	BDI-II Total	0.06 (0.03)	-0.01 to 0.12	0.31	-0.03 to 0.64	0.071	0.162	1.72	0.26	0.23
	49	BAI Total	0.06 (0.04)	-0.02 to 0.13	0.26	-0.08 to 0.59	0.130	0.238	1.72	0.26	0.23
Illness Concern	49	BDI-II Total	0.08 (0.03)	0.02 to 0.14	0.43	0.10 to 0.76	0.013	0.050	1.72	0.26	0.23
	49	BAI Total	0.02 (0.03)	-0.05 to 0.09	0.12	-0.22 to 0.45	0.489	0.711	1.72	0.26	0.23
Coherence	49	BDI-II Total	-0.01 (0.04)	-0.08 to 0.06	-0.07	-0.46 to 0.32	0.723	0.827	1.72	0.01	-0.04
	49	BAI Total	0.02 (0.04)	-0.06 to 0.10	0.10	-0.29 to 0.49	0.610	0.750	1.72	0.01	-0.04
Emotional Representation	49	BDI-II Total	0.08 (0.02)	0.03 to 0.12	0.53	0.21 to 0.86	0.002	0.009	1.72	0.31	0.28
	49	BAI Total	0.01 (0.03)	-0.05 to 0.06	0.03	-0.29 to 0.36	0.842	0.842	1.72	0.31	0.28

Note. B = unstandardized regression coefficient; SE = standard error; CI = confidence interval; β = standardized regression coefficient; VIF = variance inflation factor; R² = coefficient of determination. The 95% CIs are reported separately for B and β. p values are two-sided nominal p values; p FDR values were adjusted using the Benjamini-Hochberg false discovery rate procedure within each diagnostic group and predictor block. Each regression used model-specific complete cases.

Table S11. Sensitivity analysis: multiple linear regression models including all nine independent variables

Group	Outcome	N	Predictor	B (SE)	95% CI for B	β	95% CI for β	p	p FDR	VIF	R²	Adjusted R²
F2	Consequences	30	FEVER Precontemplation	0.95 (0.91)	-0.95 to 2.85	0.19	-0.19 to 0.57	0.310	0.774	2.80	0.76	0.65
	Consequences	30	FEVER Contemplation	3.28 (1.11)	0.96 to 5.60	0.55	0.16 to 0.95	0.008	0.190	2.95	0.76	0.65
	Consequences	30	FEVER Action	-0.64 (0.90)	-2.51 to 1.23	-0.12	-0.45 to 0.22	0.483	0.783	2.20	0.76	0.65
	Consequences	30	WHOQOL-BREF Physical	-0.02 (0.04)	-0.09 to 0.06	-0.08	-0.48 to 0.32	0.677	0.855	3.08	0.76	0.65

Group	Outcome	N	Predictor	B (SE)	95% CI for B	β	95% CI for β	p	p FDR	VIF	R²	Adjusted R²
	Consequences	30	WHOQOL-BREF Psychological	-0.10 (0.03)	-0.17 to -0.04	-0.66	-1.08 to -0.24	0.004	0.190	3.41	0.76	0.65
	Consequences	30	WHOQOL-BREF Social	-0.01 (0.02)	-0.05 to 0.03	-0.07	-0.34 to 0.20	0.579	0.783	1.39	0.76	0.65
	Consequences	30	WHOQOL-BREF Environment	0.01 (0.02)	-0.04 to 0.06	0.09	-0.23 to 0.41	0.576	0.783	1.95	0.76	0.65
	Consequences	30	BDI-II Total	-0.08 (0.06)	-0.22 to 0.05	-0.24	-0.60 to 0.13	0.199	0.652	2.61	0.76	0.65
	Consequences	30	BAI Total	0.04 (0.03)	-0.02 to 0.11	0.20	-0.11 to 0.50	0.199	0.652	1.81	0.76	0.65
	Timeline	29	FEVER Precontemplation	1.66 (1.39)	-1.26 to 4.58	0.32	-0.25 to 0.89	0.249	0.746	2.76	0.49	0.25
	Timeline	29	FEVER Contemplation	1.18 (1.69)	-2.36 to 4.72	0.20	-0.40 to 0.79	0.494	0.783	3.01	0.49	0.25
	Timeline	29	FEVER Action	-1.22 (1.38)	-4.11 to 1.67	-0.22	-0.73 to 0.30	0.389	0.783	2.23	0.49	0.25
	Timeline	29	WHOQOL-BREF Physical	-0.10 (0.06)	-0.23 to 0.02	-0.49	-1.10 to 0.12	0.106	0.547	3.16	0.49	0.25
	Timeline	29	WHOQOL-BREF Psychological	-0.02 (0.05)	-0.12 to 0.08	-0.13	-0.78 to 0.51	0.670	0.855	3.50	0.49	0.25
	Timeline	29	WHOQOL-BREF Social	-0.02 (0.03)	-0.08 to 0.04	-0.15	-0.56 to 0.25	0.441	0.783	1.41	0.49	0.25
	Timeline	29	WHOQOL-BREF Environment	0.03 (0.04)	-0.05 to 0.11	0.19	-0.32 to 0.70	0.442	0.783	2.22	0.49	0.25
	Timeline	29	BDI-II Total	-0.11 (0.10)	-0.31 to 0.09	-0.30	-0.85 to 0.25	0.272	0.754	2.61	0.49	0.25
	Timeline	29	BAI Total	0.03 (0.05)	-0.08 to 0.13	0.12	-0.34 to 0.58	0.598	0.783	1.82	0.49	0.25
	Personal Control	30	FEVER Precontemplation	-0.18 (1.37)	-3.03 to 2.67	-0.04	-0.71 to 0.63	0.898	0.898	2.80	0.26	-0.08
	Personal Control	30	FEVER Contemplation	-0.99 (1.67)	-4.47 to 2.49	-0.20	-0.89 to 0.49	0.560	0.783	2.95	0.26	-0.08
	Personal Control	30	FEVER Action	0.79 (1.34)	-2.01 to 3.60	0.17	-0.43 to 0.76	0.561	0.783	2.20	0.26	-0.08
	Personal Control	30	WHOQOL-BREF Physical	-0.02 (0.06)	-0.13 to 0.10	-0.10	-0.81 to 0.61	0.770	0.867	3.08	0.26	-0.08
	Personal Control	30	WHOQOL-BREF Psychological	0.08 (0.05)	-0.02 to 0.18	0.62	-0.12 to 1.36	0.097	0.547	3.41	0.26	-0.08
	Personal Control	30	WHOQOL-BREF Social	0.01 (0.03)	-0.05 to 0.06	0.07	-0.41 to 0.54	0.764	0.867	1.39	0.26	-0.08
	Personal Control	30	WHOQOL-BREF Environment	-0.06 (0.04)	-0.13 to 0.02	-0.44	-1.00 to 0.12	0.115	0.550	1.95	0.26	-0.08
	Personal Control	30	BDI-II Total	0.08 (0.09)	-0.12 to 0.28	0.27	-0.38 to 0.92	0.394	0.783	2.61	0.26	-0.08
	Personal Control	30	BAI Total	-0.04 (0.05)	-0.14 to 0.06	-0.21	-0.75 to 0.33	0.421	0.783	1.81	0.26	-0.08
	Treatment Control	30	FEVER Precontemplation	1.22 (1.17)	-1.23 to 3.67	0.33	-0.33 to 0.99	0.312	0.774	2.80	0.28	-0.05
	Treatment Control	30	FEVER Contemplation	0.33	-2.66 to	0.08	-0.61 to	0.820	0.881	2.95	0.28	-0.05

Group	Outcome	N	Predictor	B (SE)	95% CI for B	β	95% CI for β	p	p FDR	VIF	R²	Adjusted R²
				(1.43)	3.32		0.76					
	Treatment Control	30	FEVER Action	0.63 (1.16)	-1.78 to 3.04	0.15	-0.43 to 0.74	0.590	0.783	2.20	0.28	-0.05
	Treatment Control	30	WHOQOL-BREF Physical	0.01 (0.05)	-0.09 to 0.11	0.06	-0.64 to 0.76	0.860	0.897	3.08	0.28	-0.05
	Treatment Control	30	WHOQOL-BREF Psychological	0.02 (0.04)	-0.06 to 0.11	0.21	-0.52 to 0.95	0.550	0.783	3.41	0.28	-0.05
	Treatment Control	30	WHOQOL-BREF Social	0.02 (0.02)	-0.03 to 0.06	0.15	-0.32 to 0.62	0.510	0.783	1.39	0.28	-0.05
	Treatment Control	30	WHOQOL-BREF Environment	-0.03 (0.03)	-0.09 to 0.04	- 0.22	-0.78 to 0.33	0.408	0.783	1.95	0.28	-0.05
	Treatment Control	30	BDI-II Total	-0.02 (0.08)	-0.19 to 0.15	- 0.09	-0.73 to 0.55	0.777	0.867	2.61	0.28	-0.05
	Treatment Control	30	BAI Total	0.03 (0.04)	-0.06 to 0.12	0.17	-0.36 to 0.71	0.508	0.783	1.81	0.28	-0.05
	Identity	30	FEVER Precontemplation	0.32 (1.08)	-1.93 to 2.56	0.07	-0.43 to 0.57	0.773	0.867	2.80	0.59	0.41
	Identity	30	FEVER Contemplation	4.09 (1.32)	1.35 to 6.84	0.76	0.25 to 1.27	0.006	0.190	2.95	0.59	0.41
	Identity	30	FEVER Action	-2.13 (1.06)	-4.35 to 0.08	- 0.43	-0.87 to 0.02	0.058	0.521	2.20	0.59	0.41
	Identity	30	WHOQOL-BREF Physical	-0.03 (0.04)	-0.12 to 0.07	- 0.15	-0.67 to 0.37	0.556	0.783	3.08	0.59	0.41
	Identity	30	WHOQOL-BREF Psychological	-0.07 (0.04)	-0.15 to 0.01	- 0.50	-1.05 to 0.05	0.072	0.547	3.41	0.59	0.41
	Identity	30	WHOQOL-BREF Social	0.03 (0.02)	-0.01 to 0.08	0.25	-0.10 to 0.61	0.146	0.609	1.39	0.59	0.41
	Identity	30	WHOQOL-BREF Environment	-0.01 (0.03)	-0.07 to 0.05	- 0.08	-0.50 to 0.33	0.691	0.857	1.95	0.59	0.41
	Identity	30	BDI-II Total	-0.16 (0.07)	-0.32 to - 0.01	- 0.50	-0.98 to - 0.02	0.043	0.444	2.61	0.59	0.41
	Identity	30	BAI Total	-0.01 (0.04)	-0.09 to 0.08	- 0.03	-0.43 to 0.37	0.886	0.898	1.81	0.59	0.41
	Illness Concern	30	FEVER Precontemplation	1.59 (1.16)	-0.83 to 4.00	0.33	-0.17 to 0.83	0.186	0.652	2.80	0.59	0.40
	Illness Concern	30	FEVER Contemplation	-1.24 (1.41)	-4.19 to 1.71	- 0.22	-0.73 to 0.30	0.392	0.783	2.95	0.59	0.40
	Illness Concern	30	FEVER Action	1.59 (1.14)	-0.79 to 3.97	0.30	-0.15 to 0.74	0.178	0.652	2.20	0.59	0.40
	Illness Concern	30	WHOQOL-BREF Physical	-0.05 (0.05)	-0.15 to 0.05	- 0.29	-0.81 to 0.24	0.272	0.754	3.08	0.59	0.40
	Illness Concern	30	WHOQOL-BREF Psychological	-0.11 (0.04)	-0.19 to - 0.02	- 0.71	-1.26 to - 0.15	0.015	0.221	3.41	0.59	0.40
	Illness Concern	30	WHOQOL-BREF Social	-0.03 (0.02)	-0.08 to 0.01	- 0.25	-0.61 to 0.10	0.152	0.609	1.39	0.59	0.40
	Illness Concern	30	WHOQOL-BREF Environment	0.05 (0.03)	-0.01 to 0.12	0.35	-0.07 to 0.77	0.100	0.547	1.95	0.59	0.40
	Illness Concern	30	BDI-II Total	-0.07 (0.08)	-0.23 to 0.10	- 0.19	-0.68 to 0.29	0.417	0.783	2.61	0.59	0.40

Group	Outcome	N	Predictor	B (SE)	95% CI for B	β	95% CI for β	p	p FDR	VIF	R²	Adjusted R²
	Illness Concern	30	BAI Total	0.05 (0.04)	-0.04 to 0.13	0.21	-0.19 to 0.62	0.287	0.766	1.81	0.59	0.40
	Coherence	30	FEVER Precontemplation	-0.16 (1.14)	-2.54 to 2.22	-0.04	-0.61 to 0.53	0.890	0.898	2.80	0.47	0.23
	Coherence	30	FEVER Contemplation	0.81 (1.39)	-2.10 to 3.72	0.16	-0.42 to 0.75	0.567	0.783	2.95	0.47	0.23
	Coherence	30	FEVER Action	0.74 (1.12)	-1.60 to 3.09	0.16	-0.34 to 0.66	0.515	0.783	2.20	0.47	0.23
	Coherence	30	WHOQOL-BREF Physical	-0.04 (0.05)	-0.14 to 0.06	-0.24	-0.83 to 0.36	0.411	0.783	3.08	0.47	0.23
	Coherence	30	WHOQOL-BREF Psychological	0.05 (0.04)	-0.03 to 0.13	0.38	-0.24 to 1.01	0.215	0.672	3.41	0.47	0.23
	Coherence	30	WHOQOL-BREF Social	0.02 (0.02)	-0.03 to 0.07	0.15	-0.25 to 0.56	0.429	0.783	1.39	0.47	0.23
	Coherence	30	WHOQOL-BREF Environment	-0.01 (0.03)	-0.07 to 0.05	-0.06	-0.54 to 0.41	0.784	0.867	1.95	0.47	0.23
	Coherence	30	BDI-II Total	-0.12 (0.08)	-0.29 to 0.04	-0.40	-0.95 to 0.14	0.139	0.609	2.61	0.47	0.23
	Coherence	30	BAI Total	-0.01 (0.04)	-0.09 to 0.08	-0.05	-0.50 to 0.41	0.834	0.883	1.81	0.47	0.23
	Emotional Representation	29	FEVER Precontemplation	1.49 (0.85)	-0.30 to 3.27	0.32	-0.06 to 0.71	0.097	0.547	2.73	0.76	0.65
	Emotional Representation	29	FEVER Contemplation	0.27 (1.04)	-1.90 to 2.45	0.05	-0.34 to 0.44	0.795	0.867	2.82	0.76	0.65
	Emotional Representation	29	FEVER Action	-0.61 (0.84)	-2.37 to 1.14	-0.12	-0.47 to 0.22	0.472	0.783	2.19	0.76	0.65
	Emotional Representation	29	WHOQOL-BREF Physical	0.01 (0.04)	-0.06 to 0.08	0.06	-0.35 to 0.47	0.768	0.867	3.12	0.76	0.65
	Emotional Representation	29	WHOQOL-BREF Psychological	-0.08 (0.03)	-0.15 to -0.02	-0.58	-1.01 to -0.14	0.012	0.218	3.47	0.76	0.65
	Emotional Representation	29	WHOQOL-BREF Social	0.01 (0.02)	-0.03 to 0.04	0.04	-0.23 to 0.31	0.773	0.867	1.34	0.76	0.65
	Emotional Representation	29	WHOQOL-BREF Environment	0.05 (0.02)	0.00 to 0.10	0.35	0.02 to 0.69	0.039	0.444	2.03	0.76	0.65
	Emotional Representation	29	BDI-II Total	0.11 (0.06)	-0.02 to 0.24	0.32	-0.05 to 0.70	0.086	0.547	2.56	0.76	0.65
	Emotional Representation	29	BAI Total	0.02 (0.03)	-0.05 to 0.08	0.08	-0.23 to 0.40	0.590	0.783	1.85	0.76	0.65
	Consequences	40	FEVER Precontemplation	-0.11 (0.65)	-1.43 to 1.21	-0.04	-0.53 to 0.45	0.862	0.929	2.76	0.38	0.20
	Consequences	40	FEVER Contemplation	0.72 (0.69)	-0.69 to 2.13	0.27	-0.26 to 0.81	0.306	0.760	3.33	0.38	0.20
F3	Consequences	40	FEVER Action	-0.15 (0.63)	-1.44 to 1.14	-0.06	-0.59 to 0.46	0.809	0.929	3.19	0.38	0.20
	Consequences	40	WHOQOL-BREF Physical	-0.02 (0.02)	-0.06 to 0.02	-0.22	-0.70 to 0.26	0.362	0.766	2.68	0.38	0.20
	Consequences	40	WHOQOL-BREF Psychological	-0.03 (0.02)	-0.08 to 0.02	-0.36	-0.94 to 0.22	0.215	0.745	3.90	0.38	0.20
	Consequences	40	WHOQOL-BREF Social	-0.00	-0.03 to	-	-0.44 to	0.903	0.942	1.99	0.38	0.20

Group	Outcome	N	Predictor	B (SE)	95% CI for B	β	95% CI for β	p	p FDR	VIF	R²	Adjusted R²
				(0.02)	0.03	0.02	0.39					
	Consequences	40	WHOQOL-BREF Environment	0.01 (0.02)	-0.02 to 0.05	0.11	-0.24 to 0.46	0.535	0.897	1.45	0.38	0.20
	Consequences	40	BDI-II Total	0.04 (0.03)	-0.03 to 0.11	0.29	-0.24 to 0.81	0.272	0.745	3.21	0.38	0.20
	Consequences	40	BAI Total	-0.03 (0.04)	-0.11 to 0.05	- 0.23	-0.78 to 0.32	0.399	0.766	3.48	0.38	0.20
	Timeline	38	FEVER Precontemplation	-2.19 (1.00)	-4.24 to - 0.15	- 0.51	-0.99 to - 0.03	0.037	0.718	2.78	0.45	0.28
	Timeline	38	FEVER Contemplation	0.55 (1.07)	-1.64 to 2.74	0.13	-0.39 to 0.66	0.610	0.914	3.37	0.45	0.28
	Timeline	38	FEVER Action	1.83 (1.00)	-0.21 to 3.87	0.47	-0.05 to 0.99	0.077	0.745	3.32	0.45	0.28
	Timeline	38	WHOQOL-BREF Physical	-0.03 (0.03)	-0.10 to 0.04	- 0.20	-0.65 to 0.24	0.359	0.766	2.44	0.45	0.28
	Timeline	38	WHOQOL-BREF Psychological	-0.04 (0.04)	-0.12 to 0.04	- 0.30	-0.84 to 0.25	0.270	0.745	3.62	0.45	0.28
	Timeline	38	WHOQOL-BREF Social	-0.01 (0.03)	-0.06 to 0.04	- 0.09	-0.51 to 0.34	0.684	0.929	2.20	0.45	0.28
	Timeline	38	WHOQOL-BREF Environment	0.01 (0.03)	-0.05 to 0.06	0.06	-0.28 to 0.40	0.741	0.929	1.41	0.45	0.28
	Timeline	38	BDI-II Total	0.08 (0.05)	-0.03 to 0.19	0.35	-0.13 to 0.82	0.148	0.745	2.78	0.45	0.28
	Timeline	38	BAI Total	-0.07 (0.06)	-0.19 to 0.05	- 0.29	-0.80 to 0.22	0.253	0.745	3.14	0.45	0.28
	Personal Control	40	FEVER Precontemplation	-0.42 (0.97)	-2.39 to 1.56	- 0.10	-0.56 to 0.36	0.670	0.929	2.76	0.45	0.28
	Personal Control	40	FEVER Contemplation	0.55 (1.03)	-1.56 to 2.66	0.13	-0.37 to 0.64	0.600	0.914	3.33	0.45	0.28
	Personal Control	40	FEVER Action	1.05 (0.95)	-0.88 to 2.99	0.27	-0.23 to 0.77	0.274	0.745	3.19	0.45	0.28
	Personal Control	40	WHOQOL-BREF Physical	0.01 (0.03)	-0.06 to 0.07	0.04	-0.41 to 0.50	0.841	0.929	2.68	0.45	0.28
	Personal Control	40	WHOQOL-BREF Psychological	0.02 (0.04)	-0.06 to 0.10	0.14	-0.40 to 0.69	0.593	0.914	3.90	0.45	0.28
	Personal Control	40	WHOQOL-BREF Social	0.01 (0.02)	-0.03 to 0.06	0.11	-0.28 to 0.50	0.566	0.914	1.99	0.45	0.28
	Personal Control	40	WHOQOL-BREF Environment	-0.03 (0.03)	-0.09 to 0.02	- 0.22	-0.56 to 0.11	0.183	0.745	1.45	0.45	0.28
	Personal Control	40	BDI-II Total	-0.07 (0.05)	-0.18 to 0.03	- 0.35	-0.85 to 0.15	0.160	0.745	3.21	0.45	0.28
	Personal Control	40	BAI Total	-0.02 (0.06)	-0.13 to 0.10	- 0.07	-0.58 to 0.45	0.794	0.929	3.48	0.45	0.28
	Treatment Control	40	FEVER Precontemplation	-0.08 (1.01)	-2.14 to 1.98	- 0.02	-0.54 to 0.50	0.938	0.965	2.76	0.31	0.10
	Treatment Control	40	FEVER Contemplation	1.69 (1.08)	-0.52 to 3.89	0.43	-0.13 to 1.00	0.128	0.745	3.33	0.31	0.10
	Treatment Control	40	FEVER Action	-0.45 (0.99)	-2.47 to 1.56	- 0.12	-0.68 to 0.43	0.649	0.929	3.19	0.31	0.10

Group	Outcome	N	Predictor	B (SE)	95% CI for B	β	95% CI for β	p	p FDR	VIF	R²	Adjusted R²
	Treatment Control	40	WHOQOL-BREF Physical	-0.03 (0.03)	-0.10 to 0.04	-0.23	-0.74 to 0.28	0.362	0.766	2.68	0.31	0.10
	Treatment Control	40	WHOQOL-BREF Psychological	0.04 (0.04)	-0.04 to 0.12	0.28	-0.34 to 0.89	0.363	0.766	3.90	0.31	0.10
	Treatment Control	40	WHOQOL-BREF Social	-0.03 (0.02)	-0.08 to 0.02	-0.25	-0.69 to 0.18	0.245	0.745	1.99	0.31	0.10
	Treatment Control	40	WHOQOL-BREF Environment	0.01 (0.03)	-0.05 to 0.06	0.04	-0.33 to 0.42	0.811	0.929	1.45	0.31	0.10
	Treatment Control	40	BDI-II Total	-0.07 (0.05)	-0.18 to 0.04	-0.34	-0.89 to 0.22	0.227	0.745	3.21	0.31	0.10
	Treatment Control	40	BAI Total	0.01 (0.06)	-0.11 to 0.14	0.07	-0.51 to 0.65	0.805	0.929	3.48	0.31	0.10
	Identity	40	FEVER Precontemplation	1.11 (0.90)	-0.72 to 2.94	0.29	-0.19 to 0.76	0.224	0.745	2.76	0.41	0.24
	Identity	40	FEVER Contemplation	0.30 (0.96)	-1.66 to 2.26	0.08	-0.44 to 0.60	0.756	0.929	3.33	0.41	0.24
	Identity	40	FEVER Action	-0.64 (0.88)	-2.43 to 1.16	-0.18	-0.69 to 0.33	0.474	0.821	3.19	0.41	0.24
	Identity	40	WHOQOL-BREF Physical	-0.06 (0.03)	-0.12 to -0.00	-0.49	-0.96 to -0.02	0.040	0.718	2.68	0.41	0.24
	Identity	40	WHOQOL-BREF Psychological	-0.02 (0.03)	-0.09 to 0.05	-0.13	-0.69 to 0.44	0.646	0.929	3.90	0.41	0.24
	Identity	40	WHOQOL-BREF Social	0.01 (0.02)	-0.04 to 0.05	0.07	-0.34 to 0.47	0.735	0.929	1.99	0.41	0.24
	Identity	40	WHOQOL-BREF Environment	0.04 (0.02)	-0.01 to 0.08	0.25	-0.09 to 0.59	0.146	0.745	1.45	0.41	0.24
	Identity	40	BDI-II Total	-0.03 (0.05)	-0.12 to 0.07	-0.14	-0.65 to 0.37	0.576	0.914	3.21	0.41	0.24
	Identity	40	BAI Total	0.06 (0.05)	-0.05 to 0.16	0.28	-0.25 to 0.81	0.296	0.760	3.48	0.41	0.24
	Illness Concern	40	FEVER Precontemplation	1.09 (0.85)	-0.65 to 2.82	0.30	-0.18 to 0.78	0.211	0.745	2.76	0.39	0.21
	Illness Concern	40	FEVER Contemplation	-0.01 (0.91)	-1.86 to 1.84	-0.00	-0.53 to 0.53	0.992	0.992	3.33	0.39	0.21
	Illness Concern	40	FEVER Action	-0.11 (0.83)	-1.81 to 1.59	-0.03	-0.55 to 0.48	0.896	0.942	3.19	0.39	0.21
	Illness Concern	40	WHOQOL-BREF Physical	-0.03 (0.03)	-0.08 to 0.03	-0.22	-0.69 to 0.26	0.354	0.766	2.68	0.39	0.21
	Illness Concern	40	WHOQOL-BREF Psychological	-0.01 (0.03)	-0.07 to 0.06	-0.05	-0.62 to 0.52	0.865	0.929	3.90	0.39	0.21
	Illness Concern	40	WHOQOL-BREF Social	-0.03 (0.02)	-0.07 to 0.01	-0.28	-0.69 to 0.13	0.169	0.745	1.99	0.39	0.21
	Illness Concern	40	WHOQOL-BREF Environment	0.02 (0.02)	-0.02 to 0.07	0.19	-0.16 to 0.54	0.277	0.745	1.45	0.39	0.21
	Illness Concern	40	BDI-II Total	0.04 (0.05)	-0.05 to 0.13	0.23	-0.29 to 0.75	0.374	0.766	3.21	0.39	0.21
	Illness Concern	40	BAI Total	0.01 (0.05)	-0.09 to 0.11	0.05	-0.49 to 0.59	0.854	0.929	3.48	0.39	0.21
	Coherence	40	FEVER	-0.65	-2.48 to	-	-0.66 to	0.479	0.821	2.76	0.38	0.20

Group	Outcome	N	Predictor	B (SE)	95% CI for B	β	95% CI for β	p	p FDR	VIF	R²	Adjusted R²
			Precontemplation	(0.90)	1.19	0.17	0.32					
	Coherence	40	FEVER Contemplation	-1.76 (0.96)	-3.72 to 0.21	-0.48	-1.01 to 0.06	0.077	0.745	3.33	0.38	0.20
	Coherence	40	FEVER Action	2.86 (0.88)	1.06 to 4.66	0.83	0.31 to 1.36	0.003	0.206	3.19	0.38	0.20
	Coherence	40	WHOQOL-BREF Physical	-0.03 (0.03)	-0.09 to 0.04	-0.20	-0.68 to 0.28	0.402	0.766	2.68	0.38	0.20
	Coherence	40	WHOQOL-BREF Psychological	-0.01 (0.03)	-0.08 to 0.06	-0.07	-0.64 to 0.51	0.818	0.929	3.90	0.38	0.20
	Coherence	40	WHOQOL-BREF Social	0.02 (0.02)	-0.03 to 0.06	0.16	-0.25 to 0.57	0.440	0.812	1.99	0.38	0.20
	Coherence	40	WHOQOL-BREF Environment	0.03 (0.02)	-0.02 to 0.08	0.21	-0.14 to 0.56	0.233	0.745	1.45	0.38	0.20
	Coherence	40	BDI-II Total	0.03 (0.05)	-0.06 to 0.13	0.18	-0.34 to 0.71	0.479	0.821	3.21	0.38	0.20
	Coherence	40	BAI Total	0.01 (0.05)	-0.10 to 0.12	0.06	-0.49 to 0.60	0.832	0.929	3.48	0.38	0.20
	Emotional Representation	40	FEVER Precontemplation	-0.74 (0.67)	-2.12 to 0.63	-0.25	-0.70 to 0.21	0.279	0.745	2.76	0.46	0.30
	Emotional Representation	40	FEVER Contemplation	1.72 (0.72)	0.24 to 3.19	0.58	0.08 to 1.08	0.024	0.718	3.33	0.46	0.30
	Emotional Representation	40	FEVER Action	0.04 (0.66)	-1.31 to 1.38	0.01	-0.48 to 0.50	0.956	0.969	3.19	0.46	0.30
	Emotional Representation	40	WHOQOL-BREF Physical	-0.02 (0.02)	-0.06 to 0.03	-0.19	-0.63 to 0.26	0.404	0.766	2.68	0.46	0.30
	Emotional Representation	40	WHOQOL-BREF Psychological	-0.03 (0.03)	-0.09 to 0.02	-0.35	-0.89 to 0.19	0.192	0.745	3.90	0.46	0.30
	Emotional Representation	40	WHOQOL-BREF Social	-0.01 (0.02)	-0.04 to 0.03	-0.08	-0.47 to 0.31	0.674	0.929	1.99	0.46	0.30
	Emotional Representation	40	WHOQOL-BREF Environment	-0.00 (0.02)	-0.04 to 0.03	-0.04	-0.37 to 0.29	0.813	0.929	1.45	0.46	0.30
	Emotional Representation	40	BDI-II Total	0.05 (0.04)	-0.02 to 0.13	0.37	-0.13 to 0.86	0.139	0.745	3.21	0.46	0.30
	Emotional Representation	40	BAI Total	-0.06 (0.04)	-0.14 to 0.02	-0.38	-0.89 to 0.13	0.136	0.745	3.48	0.46	0.30

Note. B = unstandardized regression coefficient; SE = standard error; CI = confidence interval; β = standardized regression coefficient; VIF = variance inflation factor; R² = coefficient of determination. The 95% CIs are reported separately for B and β. p values are two-sided nominal p values; p FDR values were adjusted using the Benjamini-Hochberg false discovery rate procedure within each diagnostic group and predictor block. Each regression used model-specific complete cases. All nine independent variables were entered simultaneously in each model. Because complete-case sample sizes may be small, these models are presented as exploratory sensitivity analyses rather than replacements for the primary block-specific models.
