# Supplementary material for: Comparison of factors of the subjective psychological condition of hospitalized individuals with schizophrenia spectrum and other psychotic disorders, or affective disorders: associations between illness perception, quality of life, affective and anxiety symptom severity, and readiness to change
Source: Front Psychol. 2026 Oct 2;17:1829834. doi: 10.3389/fpsyg.2026.1829834 (PMC13672533; doi:10.3389/fpsyg.2026.1829834)
Supplement: Supplementary file 2 [file Data_Sheet_2.PDF]

Supplementary Materials 2

Contents

Table S1: Spearman correlations in the F2 sample, including unadjusted and Benjamini-Hochberg FDR-adjusted p-values 2
Table S2: Spearman correlations in the F3 sample, including unadjusted and Benjamini-Hochberg FDR-adjusted p-values 12
Table S3: Overview of the supplementary correlation heatmaps. 22
Figure S1: F2 Spearman correlation heatmap, including non-significant correlations (p > 0.05) 23
Figure S2: F2 Spearman correlation heatmap with FDR correction 24
Figure S3: F3 Spearman correlation heatmap, including non-significant correlations (p > 0.05) 25
Figure S4: F3 Spearman correlation heatmap with FDR correction 26

Table S1: Spearman correlations in the F2 sample, including unadjusted and Benjamini-Hochberg FDR-adjusted p-values

Variable 1	Variable 2	Spearman rho	Pairwise N	Unadjusted p	FDR-adjusted p	p < .05	FDR p < .05
B-BAI - Total	B-BDI - Total	0.43	34	0.01093	0.03014	Yes	Yes
B-BAI - Total	B-BIPQ - Coherence	-0.16	33	0.36865	0.46695	No	No
B-BAI - Total	B-BIPQ - Consequences	0.39	33	0.02440	0.05638	Yes	No
B-BAI - Total	B-BIPQ - Emotional representation	0.17	32	0.34108	0.44865	No	No
B-BAI - Total	B-BIPQ - Identity	0.17	33	0.35583	0.45927	No	No
B-BAI - Total	B-BIPQ - Illness concern	0.20	33	0.26016	0.35877	No	No
B-BAI - Total	B-BIPQ - Personal control	-0.17	33	0.34594	0.45157	No	No
B-BAI - Total	B-BIPQ - Timeline	0.08	32	0.65524	0.72259	No	No
B-BAI - Total	B-BIPQ - Total	0.32	31	0.07531	0.13700	No	No
B-BAI - Total	B-BIPQ - Treatment control	0.13	33	0.47595	0.56915	No	No
B-BAI - Total	B-WHOQOL-BREF - Environment	-0.43	34	0.01174	0.03135	Yes	Yes
B-BAI - Total	B-WHOQOL-BREF - Physical	-0.46	34	0.00582	0.01809	Yes	Yes
B-BAI - Total	B-WHOQOL-BREF - Psychological	-0.29	34	0.10006	0.17459	No	No
B-BAI - Total	B-WHOQOL-BREF - Social	-0.23	34	0.18508	0.27762	No	No
B-BAI - Total	B-WHOQOL-BREF - Total	-0.28	33	0.11177	0.18202	No	No
B-BDI - Total	B-BIPQ - Coherence	-0.31	48	0.03182	0.06802	Yes	No
B-BDI - Total	B-BIPQ - Consequences	0.49	48	< 0.001	0.00164	Yes	Yes
B-BDI - Total	B-BIPQ - Emotional representation	0.62	47	< 0.001	< 0.001	Yes	Yes
B-BDI - Total	B-BIPQ - Identity	0.29	47	0.05119	0.10061	No	No
B-BDI - Total	B-BIPQ - Illness concern	0.35	48	0.01412	0.03603	Yes	Yes
B-BDI - Total	B-BIPQ - Personal control	-0.16	47	0.29171	0.38669	No	No
B-BDI - Total	B-BIPQ - Timeline	0.19	46	0.20733	0.30303	No	No
B-BDI - Total	B-BIPQ - Total	0.50	44	< 0.001	0.00205	Yes	Yes
B-BDI - Total	B-BIPQ - Treatment control	0.03	47	0.86573	0.87598	No	No

Variable 1	Variable 2	Spearman rho	Pairwise N	Unadjusted p	FDR-adjusted p	p < .05	FDR p < .05
B-BDI - Total	B-WHOQOL-BREF - Environment	-0.38	48	0.00730	0.02189	Yes	Yes
B-BDI - Total	B-WHOQOL-BREF - Physical	-0.46	48	< 0.001	0.00357	Yes	Yes
B-BDI - Total	B-WHOQOL-BREF - Psychological	-0.73	48	< 0.001	< 0.001	Yes	Yes
B-BDI - Total	B-WHOQOL-BREF - Social	-0.29	48	0.04512	0.08971	Yes	No
B-BDI - Total	B-WHOQOL-BREF - Total	-0.43	47	0.00256	0.00860	Yes	Yes
B-BIPQ - Coherence	B-BIPQ - Consequences	-0.22	105	0.02420	0.05638	Yes	No
B-BIPQ - Coherence	B-BIPQ - Identity	-0.03	106	0.73205	0.77272	No	No
B-BIPQ - Coherence	B-BIPQ - Illness concern	-0.06	107	0.57181	0.66202	No	No
B-BIPQ - Coherence	B-BIPQ - Personal control	0.40	106	< 0.001	< 0.001	Yes	Yes
B-BIPQ - Coherence	B-BIPQ - Timeline	-0.02	102	0.82238	0.84982	No	No
B-BIPQ - Coherence	B-BIPQ - Total	-0.38	100	< 0.001	< 0.001	Yes	Yes
B-BIPQ - Coherence	B-BIPQ - Treatment control	0.21	105	0.03121	0.06799	Yes	No
B-BIPQ - Consequences	B-BIPQ - Total	0.78	100	< 0.001	< 0.001	Yes	Yes
B-BIPQ - Emotional representation	B-BIPQ - Coherence	-0.24	105	0.01565	0.03823	Yes	Yes
B-BIPQ - Emotional representation	B-BIPQ - Consequences	0.57	104	< 0.001	< 0.001	Yes	Yes
B-BIPQ - Emotional representation	B-BIPQ - Identity	0.56	105	< 0.001	< 0.001	Yes	Yes
B-BIPQ - Emotional representation	B-BIPQ - Illness concern	0.54	105	< 0.001	< 0.001	Yes	Yes
B-BIPQ - Emotional representation	B-BIPQ - Personal control	-0.15	105	0.13020	0.20808	No	No
B-BIPQ - Emotional	B-BIPQ - Timeline	0.42	101	< 0.001	< 0.001	Yes	Yes

Variable 1	Variable 2	Spearman rho	Pairwise N	Unadjusted p	FDR-adjusted p	p < .05	FDR p < .05
representation							
B-BIPQ - Emotional representation	B-BIPQ - Total	0.73	100	< 0.001	< 0.001	Yes	Yes
B-BIPQ - Emotional representation	B-BIPQ - Treatment control	0.06	104	0.57526	0.66202	No	No
B-BIPQ - Identity	B-BIPQ - Consequences	0.75	104	< 0.001	< 0.001	Yes	Yes
B-BIPQ - Identity	B-BIPQ - Personal control	-0.16	106	0.09735	0.17162	No	No
B-BIPQ - Identity	B-BIPQ - Timeline	0.41	102	< 0.001	< 0.001	Yes	Yes
B-BIPQ - Identity	B-BIPQ - Total	0.72	100	< 0.001	< 0.001	Yes	Yes
B-BIPQ - Identity	B-BIPQ - Treatment control	0.07	105	0.45057	0.54748	No	No
B-BIPQ - Illness concern	B-BIPQ - Consequences	0.65	105	< 0.001	< 0.001	Yes	Yes
B-BIPQ - Illness concern	B-BIPQ - Identity	0.65	106	< 0.001	< 0.001	Yes	Yes
B-BIPQ - Illness concern	B-BIPQ - Personal control	-0.11	106	0.28403	0.38244	No	No
B-BIPQ - Illness concern	B-BIPQ - Timeline	0.25	102	0.01191	0.03135	Yes	Yes
B-BIPQ - Illness concern	B-BIPQ - Total	0.64	100	< 0.001	< 0.001	Yes	Yes
B-BIPQ - Illness concern	B-BIPQ - Treatment control	0.02	105	0.87398	0.87912	No	No
B-BIPQ - Personal control	B-BIPQ - Consequences	-0.24	104	0.01256	0.03255	Yes	Yes
B-BIPQ - Personal control	B-BIPQ - Timeline	-0.19	102	0.05809	0.11160	No	No
B-BIPQ - Personal control	B-BIPQ - Total	-0.49	100	< 0.001	< 0.001	Yes	Yes
B-BIPQ - Timeline	B-BIPQ - Consequences	0.40	101	< 0.001	< 0.001	Yes	Yes
B-BIPQ - Timeline	B-BIPQ - Total	0.59	100	< 0.001	< 0.001	Yes	Yes
B-BIPQ -	B-BIPQ -	0.13	104	0.19944	0.29467	No	No

Variable 1	Variable 2	Spearman rho	Pairwise N	Unadjusted p	FDR-adjusted p	p < .05	FDR p < .05
Treatment control	Consequences						
B-BIPQ - Treatment control	B-BIPQ - Personal control	0.50	105	< 0.001	< 0.001	Yes	Yes
B-BIPQ - Treatment control	B-BIPQ - Timeline	0.02	102	0.83462	0.85461	No	No
B-BIPQ - Treatment control	B-BIPQ - Total	-0.16	100	0.11512	0.18571	No	No
B-FEVER - Action	B-BAI - Total	-0.09	32	0.60507	0.68521	No	No
B-FEVER - Action	B-BDI - Total	-0.14	41	0.39980	0.49903	No	No
B-FEVER - Action	B-BIPQ - Coherence	0.27	94	0.00785	0.02315	Yes	Yes
B-FEVER - Action	B-BIPQ - Consequences	0.08	92	0.42440	0.52211	No	No
B-FEVER - Action	B-BIPQ - Emotional representation	0.19	93	0.06279	0.11930	No	No
B-FEVER - Action	B-BIPQ - Identity	0.19	94	0.06905	0.12975	No	No
B-FEVER - Action	B-BIPQ - Illness concern	0.20	94	0.05415	0.10522	No	No
B-FEVER - Action	B-BIPQ - Personal control	0.17	94	0.10323	0.17652	No	No
B-FEVER - Action	B-BIPQ - Timeline	0.05	90	0.61388	0.68610	No	No
B-FEVER - Action	B-BIPQ - Total	0.08	88	0.45143	0.54748	No	No
B-FEVER - Action	B-BIPQ - Treatment control	0.29	93	0.00510	0.01616	Yes	Yes
B-FEVER - Action	B-FEVER - Contemplation	0.64	92	< 0.001	< 0.001	Yes	Yes
B-FEVER - Action	B-FEVER - Precontemplation	0.12	88	0.25824	0.35877	No	No
B-FEVER - Action	B-WHOQOL-BREF - Environment	0.39	43	0.01064	0.02983	Yes	Yes
B-FEVER - Action	B-WHOQOL-BREF - Physical	0.32	43	0.03513	0.07416	Yes	No
B-FEVER - Action	B-WHOQOL-BREF - Psychological	0.21	43	0.17988	0.27463	No	No

Variable 1	Variable 2	Spearman rho	Pairwise N	Unadjusted p	FDR-adjusted p	p < .05	FDR p < .05
B-FEVER - Action	B-WHOQOL-BREF - Social	0.23	43	0.14448	0.22605	No	No
B-FEVER - Action	B-WHOQOL-BREF - Total	0.18	42	0.25556	0.35877	No	No
B-FEVER - Contemplation	B-BAI - Total	0.22	32	0.22891	0.33172	No	No
B-FEVER - Contemplation	B-BDI - Total	0.17	42	0.27075	0.37039	No	No
B-FEVER - Contemplation	B-BIPQ - Coherence	0.02	93	0.85993	0.87529	No	No
B-FEVER - Contemplation	B-BIPQ - Consequences	0.33	91	0.00165	0.00589	Yes	Yes
B-FEVER - Contemplation	B-BIPQ - Emotional representation	0.30	92	0.00335	0.01103	Yes	Yes
B-FEVER - Contemplation	B-BIPQ - Identity	0.23	93	0.02992	0.06685	Yes	No
B-FEVER - Contemplation	B-BIPQ - Illness concern	0.25	93	0.01511	0.03800	Yes	Yes
B-FEVER - Contemplation	B-BIPQ - Personal control	0.02	93	0.82497	0.84982	No	No
B-FEVER - Contemplation	B-BIPQ - Timeline	0.06	91	0.55076	0.65402	No	No
B-FEVER - Contemplation	B-BIPQ - Total	0.22	89	0.03940	0.08118	Yes	No
B-FEVER - Contemplation	B-BIPQ - Treatment control	0.19	92	0.07281	0.13533	No	No
B-FEVER - Contemplation	B-FEVER - Precontemplation	0.32	88	0.00230	0.00804	Yes	Yes
B-FEVER - Contemplation	B-WHOQOL-BREF - Environment	0.25	43	0.10768	0.17876	No	No
B-FEVER - Contemplation	B-WHOQOL-BREF - Physical	0.09	43	0.57685	0.66202	No	No
B-FEVER - Contemplation	B-WHOQOL-BREF - Psychological	-0.09	43	0.57238	0.66202	No	No
B-FEVER - Contemplation	B-WHOQOL-BREF - Social	0.06	43	0.72297	0.76787	No	No
B-FEVER - Contemplation	B-WHOQOL-BREF - Total	-0.04	42	0.77811	0.81132	No	No
B-FEVER - Precontemplation	B-BAI - Total	0.07	31	0.69893	0.75168	No	No
B-FEVER - Precontemplati	B-BDI - Total	0.34	40	0.03010	0.06685	Yes	No

Variable 1	Variable 2	Spearman rho	Pairwise N	Unadjusted p	FDR-adjusted p	p < .05	FDR p < .05
on							
B-FEVER - Precontemplation	B-BIPQ - Coherence	-0.04	88	0.71875	0.76787	No	No
B-FEVER - Precontemplation	B-BIPQ - Consequences	0.41	86	< 0.001	< 0.001	Yes	Yes
B-FEVER - Precontemplation	B-BIPQ - Emotional representation	0.27	87	0.01192	0.03135	Yes	Yes
B-FEVER - Precontemplation	B-BIPQ - Identity	0.22	88	0.04371	0.08898	Yes	No
B-FEVER - Precontemplation	B-BIPQ - Illness concern	0.28	88	0.00947	0.02699	Yes	Yes
B-FEVER - Precontemplation	B-BIPQ - Personal control	-0.03	88	0.76976	0.80754	No	No
B-FEVER - Precontemplation	B-BIPQ - Timeline	0.23	86	0.03141	0.06799	Yes	No
B-FEVER - Precontemplation	B-BIPQ - Total	0.31	84	0.00420	0.01354	Yes	Yes
B-FEVER - Precontemplation	B-BIPQ - Treatment control	0.15	87	0.17903	0.27463	No	No
B-FEVER - Precontemplation	B-WHOQOL-BREF - Environment	-0.07	41	0.65219	0.72259	No	No
B-FEVER - Precontemplation	B-WHOQOL-BREF - Physical	-0.25	41	0.10896	0.17915	No	No
B-FEVER - Precontemplation	B-WHOQOL-BREF - Psychological	-0.07	41	0.65920	0.72259	No	No
B-FEVER - Precontemplation	B-WHOQOL-BREF - Social	0.00	41	0.99432	0.99432	No	No
B-FEVER - Precontemplation	B-WHOQOL-BREF - Total	-0.21	40	0.19990	0.29467	No	No
B-WHOQOL-	B-BIPQ - Coherence	0.26	49	0.07489	0.13700	No	No

Variable 1	Variable 2	Spearman rho	Pairwise N	Unadjusted p	FDR-adjusted p	p < .05	FDR p < .05
BREF - Environment							
B-WHOQOL-BREF - Environment	B-BIPQ - Consequences	-0.32	48	0.02594	0.05914	Yes	No
B-WHOQOL-BREF - Environment	B-BIPQ - Emotional representation	-0.17	47	0.25455	0.35877	No	No
B-WHOQOL-BREF - Environment	B-BIPQ - Identity	-0.26	48	0.07681	0.13827	No	No
B-WHOQOL-BREF - Environment	B-BIPQ - Illness concern	-0.21	49	0.14541	0.22605	No	No
B-WHOQOL-BREF - Environment	B-BIPQ - Personal control	0.06	48	0.67330	0.72870	No	No
B-WHOQOL-BREF - Environment	B-BIPQ - Timeline	-0.24	46	0.10733	0.17876	No	No
B-WHOQOL-BREF - Environment	B-BIPQ - Total	-0.25	44	0.10110	0.17462	No	No
B-WHOQOL-BREF - Environment	B-BIPQ - Treatment control	0.08	48	0.57055	0.66202	No	No
B-WHOQOL-BREF - Environment	B-WHOQOL-BREF - Physical	0.67	50	< 0.001	< 0.001	Yes	Yes
B-WHOQOL-BREF - Environment	B-WHOQOL-BREF - Psychological	0.50	50	< 0.001	< 0.001	Yes	Yes
B-WHOQOL-BREF - Environment	B-WHOQOL-BREF - Social	0.55	50	< 0.001	< 0.001	Yes	Yes
B-WHOQOL-BREF - Environment	B-WHOQOL-BREF - Total	0.58	49	< 0.001	< 0.001	Yes	Yes
B-WHOQOL-BREF - Physical	B-BIPQ - Coherence	0.24	49	0.09538	0.16990	No	No
B-WHOQOL-BREF - Physical	B-BIPQ - Consequences	-0.54	48	< 0.001	< 0.001	Yes	Yes

Variable 1	Variable 2	Spearman rho	Pairwise N	Unadjusted p	FDR-adjusted p	p < .05	FDR p < .05
B-WHOQOL-BREF - Physical	B-BIPQ - Emotional representation	-0.45	47	0.00141	0.00514	Yes	Yes
B-WHOQOL-BREF - Physical	B-BIPQ - Identity	-0.38	48	0.00860	0.02494	Yes	Yes
B-WHOQOL-BREF - Physical	B-BIPQ - Illness concern	-0.49	49	< 0.001	0.00163	Yes	Yes
B-WHOQOL-BREF - Physical	B-BIPQ - Personal control	0.17	48	0.25354	0.35877	No	No
B-WHOQOL-BREF - Physical	B-BIPQ - Timeline	-0.39	46	0.00722	0.02189	Yes	Yes
B-WHOQOL-BREF - Physical	B-BIPQ - Total	-0.52	44	< 0.001	0.00145	Yes	Yes
B-WHOQOL-BREF - Physical	B-BIPQ - Treatment control	0.08	48	0.59156	0.67437	No	No
B-WHOQOL-BREF - Physical	B-WHOQOL-BREF - Total	0.65	49	< 0.001	< 0.001	Yes	Yes
B-WHOQOL-BREF - Psychological	B-BIPQ - Coherence	0.34	49	0.01542	0.03822	Yes	Yes
B-WHOQOL-BREF - Psychological	B-BIPQ - Consequences	-0.59	48	< 0.001	< 0.001	Yes	Yes
B-WHOQOL-BREF - Psychological	B-BIPQ - Emotional representation	-0.63	47	< 0.001	< 0.001	Yes	Yes
B-WHOQOL-BREF - Psychological	B-BIPQ - Identity	-0.43	48	0.00241	0.00825	Yes	Yes
B-WHOQOL-BREF - Psychological	B-BIPQ - Illness concern	-0.52	49	< 0.001	< 0.001	Yes	Yes
B-WHOQOL-BREF - Psychological	B-BIPQ - Personal control	0.29	48	0.04425	0.08903	Yes	No
B-WHOQOL-BREF -	B-BIPQ - Timeline	-0.35	46	0.01761	0.04241	Yes	Yes

Variable 1	Variable 2	Spearman rho	Pairwise N	Unadjusted p	FDR-adjusted p	p < .05	FDR p < .05
Psychological							
B-WHOQOL-BREF - Psychological	B-BIPQ - Total	-0.64	44	< 0.001	< 0.001	Yes	Yes
B-WHOQOL-BREF - Psychological	B-BIPQ - Treatment control	0.16	48	0.27365	0.37138	No	No
B-WHOQOL-BREF - Psychological	B-WHOQOL-BREF - Physical	0.58	50	< 0.001	< 0.001	Yes	Yes
B-WHOQOL-BREF - Psychological	B-WHOQOL-BREF - Total	0.64	49	< 0.001	< 0.001	Yes	Yes
B-WHOQOL-BREF - Social	B-BIPQ - Coherence	0.19	49	0.18232	0.27591	No	No
B-WHOQOL-BREF - Social	B-BIPQ - Consequences	-0.13	48	0.36066	0.46025	No	No
B-WHOQOL-BREF - Social	B-BIPQ - Emotional representation	-0.12	47	0.42225	0.52211	No	No
B-WHOQOL-BREF - Social	B-BIPQ - Identity	0.06	48	0.66853	0.72814	No	No
B-WHOQOL-BREF - Social	B-BIPQ - Illness concern	-0.15	49	0.28961	0.38669	No	No
B-WHOQOL-BREF - Social	B-BIPQ - Personal control	0.11	48	0.46668	0.56199	No	No
B-WHOQOL-BREF - Social	B-BIPQ - Timeline	-0.08	46	0.61191	0.68610	No	No
B-WHOQOL-BREF - Social	B-BIPQ - Total	-0.14	44	0.35721	0.45927	No	No
B-WHOQOL-BREF - Social	B-BIPQ - Treatment control	0.13	48	0.38616	0.48554	No	No
B-WHOQOL-BREF - Social	B-WHOQOL-BREF - Physical	0.45	50	0.00103	0.00400	Yes	Yes
B-WHOQOL-BREF - Social	B-WHOQOL-BREF - Psychological	0.30	50	0.03649	0.07610	Yes	No
B-WHOQOL-BREF - Social	B-WHOQOL-BREF - Total	0.32	49	0.02381	0.05638	Yes	No
B-WHOQOL-BREF - Total	B-BIPQ - Coherence	0.24	48	0.10622	0.17876	No	No
B-WHOQOL-BREF - Total	B-BIPQ - Consequences	-0.57	47	< 0.001	< 0.001	Yes	Yes
B-WHOQOL-BREF - Total	B-BIPQ - Emotional representation	-0.47	46	0.00111	0.00422	Yes	Yes

Variable 1	Variable 2	Spearman rho	Pairwise N	Unadjusted p	FDR-adjusted p	p < .05	FDR p < .05
B-WHOQOL-BREF - Total	B-BIPQ - Identity	-0.45	47	0.00141	0.00514	Yes	Yes
B-WHOQOL-BREF - Total	B-BIPQ - Illness concern	-0.47	48	< 0.001	0.00271	Yes	Yes
B-WHOQOL-BREF - Total	B-BIPQ - Personal control	0.22	47	0.14032	0.22218	No	No
B-WHOQOL-BREF - Total	B-BIPQ - Timeline	-0.52	45	< 0.001	0.00114	Yes	Yes
B-WHOQOL-BREF - Total	B-BIPQ - Total	-0.56	43	< 0.001	< 0.001	Yes	Yes
B-WHOQOL-BREF - Total	B-BIPQ - Treatment control	0.17	47	0.25953	0.35877	No	No

Table S2: Spearman correlations in the F3 sample, including unadjusted and Benjamini-Hochberg FDR-adjusted p-values

Variable 1	Variable 2	Spearman rho	Pairwise N	Unadjusted p	FDR-adjusted p	p < .05	FDR p < .05
B-BAI - Total	B-BDI - Total	0.62	51	< 0.001	< 0.001	Yes	Yes
B-BAI - Total	B-BIPQ - Coherence	0.10	49	0.48854	0.55479	No	No
B-BAI - Total	B-BIPQ - Consequences	0.39	49	0.00498	0.01051	Yes	Yes
B-BAI - Total	B-BIPQ - Emotional representation	0.34	49	0.01609	0.02991	Yes	Yes
B-BAI - Total	B-BIPQ - Identity	0.48	49	< 0.001	0.00130	Yes	Yes
B-BAI - Total	B-BIPQ - Illness concern	0.39	49	0.00518	0.01080	Yes	Yes
B-BAI - Total	B-BIPQ - Personal control	-0.15	49	0.28766	0.39351	No	No
B-BAI - Total	B-BIPQ - Timeline	0.42	47	0.00295	0.00655	Yes	Yes
B-BAI - Total	B-BIPQ - Total	0.47	47	< 0.001	0.00208	Yes	Yes
B-BAI - Total	B-BIPQ - Treatment control	-0.12	49	0.41492	0.50260	No	No
B-BAI - Total	B-WHOQOL-BREF - Environment	-0.48	51	< 0.001	< 0.001	Yes	Yes
B-BAI - Total	B-WHOQOL-BREF - Physical	-0.68	51	< 0.001	< 0.001	Yes	Yes
B-BAI - Total	B-WHOQOL-BREF - Psychological	-0.62	51	< 0.001	< 0.001	Yes	Yes
B-BAI - Total	B-WHOQOL-BREF - Social	-0.44	51	0.00133	0.00315	Yes	Yes
B-BAI - Total	B-WHOQOL-BREF - Total	-0.33	51	0.01711	0.03147	Yes	Yes
B-BDI - Total	B-BIPQ - Coherence	-0.07	84	0.49847	0.56078	No	No
B-BDI - Total	B-BIPQ - Consequences	0.54	84	< 0.001	< 0.001	Yes	Yes
B-BDI - Total	B-BIPQ - Emotional	0.54	84	< 0.001	< 0.001	Yes	Yes

Variable 1	Variable 2	Spearman rho	Pairwise N	Unadjusted p	FDR-adjusted p	p < .05	FDR p < .05
	representation						
B-BDI - Total	B-BIPQ - Identity	0.45	83	< 0.001	< 0.001	Yes	Yes
B-BDI - Total	B-BIPQ - Illness concern	0.55	83	< 0.001	< 0.001	Yes	Yes
B-BDI - Total	B-BIPQ - Personal control	-0.43	82	< 0.001	< 0.001	Yes	Yes
B-BDI - Total	B-BIPQ - Timeline	0.49	79	< 0.001	< 0.001	Yes	Yes
B-BDI - Total	B-BIPQ - Total	0.70	77	< 0.001	< 0.001	Yes	Yes
B-BDI - Total	B-BIPQ - Treatment control	-0.35	84	< 0.001	0.00230	Yes	Yes
B-BDI - Total	B-WHOQOL-BREF - Environment	-0.36	85	< 0.001	0.00172	Yes	Yes
B-BDI - Total	B-WHOQOL-BREF - Physical	-0.56	85	< 0.001	< 0.001	Yes	Yes
B-BDI - Total	B-WHOQOL-BREF - Psychological	-0.73	85	< 0.001	< 0.001	Yes	Yes
B-BDI - Total	B-WHOQOL-BREF - Social	-0.31	85	0.00374	0.00819	Yes	Yes
B-BDI - Total	B-WHOQOL-BREF - Total	-0.37	85	< 0.001	0.00133	Yes	Yes
B-BIPQ - Coherence	B-BIPQ - Consequences	-0.09	250	0.17780	0.26439	No	No
B-BIPQ - Coherence	B-BIPQ - Identity	-0.02	248	0.77859	0.78780	No	No
B-BIPQ - Coherence	B-BIPQ - Illness concern	-0.06	249	0.34897	0.45207	No	No
B-BIPQ - Coherence	B-BIPQ - Personal control	0.21	246	< 0.001	0.00185	Yes	Yes
B-BIPQ - Coherence	B-BIPQ - Timeline	-0.11	240	0.09567	0.15009	No	No
B-BIPQ - Coherence	B-BIPQ - Total	-0.38	233	< 0.001	< 0.001	Yes	Yes
B-BIPQ - Coherence	B-BIPQ - Treatment control	0.23	247	< 0.001	< 0.001	Yes	Yes
B-BIPQ - Consequences	B-BIPQ - Total	0.73	233	< 0.001	< 0.001	Yes	Yes

Variable 1	Variable 2	Spearman rho	Pairwise N	Unadjusted p	FDR-adjusted p	p < .05	FDR p < .05
B-BIPQ - Emotional representation	B-BIPQ - Coherence	0.06	248	0.34075	0.44954	No	No
B-BIPQ - Emotional representation	B-BIPQ - Consequences	0.56	248	< 0.001	< 0.001	Yes	Yes
B-BIPQ - Emotional representation	B-BIPQ - Identity	0.54	248	< 0.001	< 0.001	Yes	Yes
B-BIPQ - Emotional representation	B-BIPQ - Illness concern	0.53	248	< 0.001	< 0.001	Yes	Yes
B-BIPQ - Emotional representation	B-BIPQ - Personal control	-0.25	244	< 0.001	< 0.001	Yes	Yes
B-BIPQ - Emotional representation	B-BIPQ - Timeline	0.37	239	< 0.001	< 0.001	Yes	Yes
B-BIPQ - Emotional representation	B-BIPQ - Total	0.62	233	< 0.001	< 0.001	Yes	Yes
B-BIPQ - Emotional representation	B-BIPQ - Treatment control	0.03	246	0.62542	0.67688	No	No
B-BIPQ - Identity	B-BIPQ - Consequences	0.60	248	< 0.001	< 0.001	Yes	Yes
B-BIPQ - Identity	B-BIPQ - Personal control	-0.26	245	< 0.001	< 0.001	Yes	Yes
B-BIPQ - Identity	B-BIPQ - Timeline	0.35	239	< 0.001	< 0.001	Yes	Yes
B-BIPQ - Identity	B-BIPQ - Total	0.65	233	< 0.001	< 0.001	Yes	Yes
B-BIPQ - Identity	B-BIPQ - Treatment control	0.11	246	0.09373	0.14979	No	No
B-BIPQ - Illness concern	B-BIPQ - Consequences	0.57	249	< 0.001	< 0.001	Yes	Yes
B-BIPQ - Illness concern	B-BIPQ - Identity	0.57	248	< 0.001	< 0.001	Yes	Yes
B-BIPQ - Illness concern	B-BIPQ - Personal control	-0.25	245	< 0.001	< 0.001	Yes	Yes
B-BIPQ - Illness concern	B-BIPQ - Timeline	0.31	240	< 0.001	< 0.001	Yes	Yes

Variable 1	Variable 2	Spearman rho	Pairwise N	Unadjusted p	FDR-adjusted p	p < .05	FDR p < .05
B-BIPQ - Illness concern	B-BIPQ - Total	0.65	233	< 0.001	< 0.001	Yes	Yes
B-BIPQ - Illness concern	B-BIPQ - Treatment control	0.04	246	0.48399	0.55479	No	No
B-BIPQ - Personal control	B-BIPQ - Consequences	-0.36	246	< 0.001	< 0.001	Yes	Yes
B-BIPQ - Personal control	B-BIPQ - Timeline	-0.22	236	< 0.001	0.00164	Yes	Yes
B-BIPQ - Personal control	B-BIPQ - Total	-0.59	233	< 0.001	< 0.001	Yes	Yes
B-BIPQ - Timeline	B-BIPQ - Consequences	0.39	240	< 0.001	< 0.001	Yes	Yes
B-BIPQ - Timeline	B-BIPQ - Total	0.65	233	< 0.001	< 0.001	Yes	Yes
B-BIPQ - Treatment control	B-BIPQ - Consequences	-0.02	247	0.72034	0.75569	No	No
B-BIPQ - Treatment control	B-BIPQ - Personal control	0.17	243	0.00856	0.01683	Yes	Yes
B-BIPQ - Treatment control	B-BIPQ - Timeline	-0.17	238	0.00924	0.01763	Yes	Yes
B-BIPQ - Treatment control	B-BIPQ - Total	-0.29	233	< 0.001	< 0.001	Yes	Yes
B-FEVER - Action	B-BAI - Total	-0.07	45	0.64884	0.69780	No	No
B-FEVER - Action	B-BDI - Total	-0.08	81	0.47253	0.54967	No	No
B-FEVER - Action	B-BIPQ - Coherence	0.19	226	0.00408	0.00871	Yes	Yes
B-FEVER - Action	B-BIPQ - Consequences	0.06	226	0.35243	0.45313	No	No
B-FEVER - Action	B-BIPQ - Emotional representation	0.19	224	0.00527	0.01086	Yes	Yes
B-FEVER - Action	B-BIPQ - Identity	0.17	224	0.00900	0.01749	Yes	Yes
B-FEVER - Action	B-BIPQ - Illness concern	0.09	225	0.19378	0.28566	No	No
B-FEVER - Action	B-BIPQ - Personal control	0.15	223	0.02952	0.05204	Yes	No
B-FEVER - Action	B-BIPQ - Timeline	0.00	217	0.98094	0.98094	No	No
B-FEVER - Action	B-BIPQ - Total	-0.08	211	0.23643	0.33691	No	No
B-FEVER - Action	B-BIPQ -	0.38	223	< 0.001	< 0.001	Yes	Yes

Variable 1	Variable 2	Spearman rho	Pairwise N	Unadjusted p	FDR-adjusted p	p < .05	FDR p < .05
	Treatment control						
B-FEVER - Action	B-FEVER - Contemplation	0.61	221	< 0.001	< 0.001	Yes	Yes
B-FEVER - Action	B-FEVER - Precontemplation	0.35	212	< 0.001	< 0.001	Yes	Yes
B-FEVER - Action	B-WHOQOL-BREF - Environment	0.13	79	0.26969	0.37191	No	No
B-FEVER - Action	B-WHOQOL-BREF - Physical	0.07	79	0.54644	0.60677	No	No
B-FEVER - Action	B-WHOQOL-BREF - Psychological	0.20	79	0.07866	0.13187	No	No
B-FEVER - Action	B-WHOQOL-BREF - Social	0.06	79	0.57269	0.63181	No	No
B-FEVER - Action	B-WHOQOL-BREF - Total	0.05	79	0.65575	0.70083	No	No
B-FEVER - Contemplation	B-BAI - Total	0.10	43	0.54335	0.60677	No	No
B-FEVER - Contemplation	B-BDI - Total	-0.05	78	0.69149	0.72990	No	No
B-FEVER - Contemplation	B-BIPQ - Coherence	0.10	222	0.14038	0.21432	No	No
B-FEVER - Contemplation	B-BIPQ - Consequences	0.08	222	0.25706	0.36030	No	No
B-FEVER - Contemplation	B-BIPQ - Emotional representation	0.20	220	0.00232	0.00529	Yes	Yes
B-FEVER - Contemplation	B-BIPQ - Identity	0.16	220	0.01929	0.03473	Yes	Yes
B-FEVER - Contemplation	B-BIPQ - Illness concern	0.13	221	0.04797	0.08285	Yes	No
B-FEVER - Contemplation	B-BIPQ - Personal control	0.02	219	0.74554	0.77265	No	No
B-FEVER - Contemplation	B-BIPQ - Timeline	-0.05	212	0.47166	0.54967	No	No
B-FEVER - Contemplation	B-BIPQ - Total	-0.06	206	0.36876	0.46614	No	No
B-FEVER - Contemplation	B-BIPQ - Treatment	0.39	219	< 0.001	< 0.001	Yes	Yes

Variable 1	Variable 2	Spearman rho	Pairwise N	Unadjusted p	FDR-adjusted p	p < .05	FDR p < .05
	control						
B-FEVER - Contemplation	B-FEVER - Precontemplation	0.30	210	< 0.001	< 0.001	Yes	Yes
B-FEVER - Contemplation	B-WHOQOL-BREF - Environment	0.09	76	0.41509	0.50260	No	No
B-FEVER - Contemplation	B-WHOQOL-BREF - Physical	0.06	76	0.59200	0.64893	No	No
B-FEVER - Contemplation	B-WHOQOL-BREF - Psychological	0.20	76	0.08488	0.13824	No	No
B-FEVER - Contemplation	B-WHOQOL-BREF - Social	0.18	76	0.11270	0.17362	No	No
B-FEVER - Contemplation	B-WHOQOL-BREF - Total	0.10	76	0.36789	0.46614	No	No
B-FEVER - Precontemplation	B-BAI - Total	-0.18	44	0.25299	0.35753	No	No
B-FEVER - Precontemplation	B-BDI - Total	0.11	75	0.32684	0.43664	No	No
B-FEVER - Precontemplation	B-BIPQ - Coherence	0.05	212	0.45862	0.54085	No	No
B-FEVER - Precontemplation	B-BIPQ - Consequences	0.12	212	0.07677	0.12997	No	No
B-FEVER - Precontemplation	B-BIPQ - Emotional representation	0.07	212	0.34175	0.44954	No	No
B-FEVER - Precontemplation	B-BIPQ - Identity	0.06	211	0.41246	0.50260	No	No
B-FEVER - Precontemplation	B-BIPQ - Illness concern	0.06	212	0.41736	0.50260	No	No
B-FEVER - Precontemplation	B-BIPQ - Personal control	-0.05	209	0.48837	0.55479	No	No
B-FEVER - Precontemplation	B-BIPQ - Timeline	0.02	203	0.73313	0.76443	No	No
B-FEVER - Precontemplation	B-BIPQ - Total	0.05	199	0.44998	0.53435	No	No
B-FEVER - Precontemplation	B-BIPQ - Treatment control	0.17	210	0.01476	0.02774	Yes	Yes
B-FEVER - Precontemplation	B-WHOQOL-BREF -	0.30	73	0.00928	0.01763	Yes	Yes

Variable 1	Variable 2	Spearman rho	Pairwise N	Unadjusted p	FDR-adjusted p	p < .05	FDR p < .05
	Environment						
B-FEVER - Precontemplation	B-WHOQOL-BREF - Physical	0.10	73	0.42268	0.50545	No	No
B-FEVER - Precontemplation	B-WHOQOL-BREF - Psychological	-0.11	73	0.37073	0.46614	No	No
B-FEVER - Precontemplation	B-WHOQOL-BREF - Social	0.21	73	0.07085	0.12115	No	No
B-FEVER - Precontemplation	B-WHOQOL-BREF - Total	0.03	73	0.77604	0.78780	No	No
B-WHOQOL-BREF - Environment	B-BIPQ - Coherence	0.14	84	0.20775	0.30363	No	No
B-WHOQOL-BREF - Environment	B-BIPQ - Consequences	-0.12	84	0.29051	0.39427	No	No
B-WHOQOL-BREF - Environment	B-BIPQ - Emotional representation	-0.15	84	0.16568	0.25072	No	No
B-WHOQOL-BREF - Environment	B-BIPQ - Identity	-0.12	84	0.26967	0.37191	No	No
B-WHOQOL-BREF - Environment	B-BIPQ - Illness concern	-0.13	83	0.23262	0.33691	No	No
B-WHOQOL-BREF - Environment	B-BIPQ - Personal control	-0.05	83	0.66585	0.70720	No	No
B-WHOQOL-BREF - Environment	B-BIPQ - Timeline	-0.19	79	0.08950	0.14439	No	No
B-WHOQOL-BREF - Environment	B-BIPQ - Total	-0.10	78	0.39562	0.49022	No	No
B-WHOQOL-BREF - Environment	B-BIPQ - Treatment control	0.12	84	0.29385	0.39566	No	No
B-WHOQOL-BREF - Environment	B-WHOQOL-BREF - Physical	0.51	87	< 0.001	< 0.001	Yes	Yes
B-WHOQOL-BREF - Environment	B-WHOQOL-BREF - Psychological	0.37	87	< 0.001	0.00114	Yes	Yes
B-WHOQOL-BREF - Environment	B-WHOQOL-BREF - Social	0.44	86	< 0.001	< 0.001	Yes	Yes
B-WHOQOL-BREF - Environment	B-WHOQOL-BREF - Total	0.29	87	0.00707	0.01422	Yes	Yes
B-WHOQOL-BREF - Physical	B-BIPQ - Coherence	-0.03	84	0.76265	0.78092	No	No
B-WHOQOL-BREF	B-BIPQ -	-0.51	84	< 0.001	< 0.001	Yes	Yes

Variable 1	Variable 2	Spearman rho	Pairwise N	Unadjusted p	FDR-adjusted p	p < .05	FDR p < .05
- Physical	Consequences						
B-WHOQOL-BREF - Physical	B-BIPQ - Emotional representation	-0.37	84	< 0.001	0.00149	Yes	Yes
B-WHOQOL-BREF - Physical	B-BIPQ - Identity	-0.58	84	< 0.001	< 0.001	Yes	Yes
B-WHOQOL-BREF - Physical	B-BIPQ - Illness concern	-0.46	83	< 0.001	< 0.001	Yes	Yes
B-WHOQOL-BREF - Physical	B-BIPQ - Personal control	0.25	83	0.02330	0.04150	Yes	Yes
B-WHOQOL-BREF - Physical	B-BIPQ - Timeline	-0.41	79	< 0.001	< 0.001	Yes	Yes
B-WHOQOL-BREF - Physical	B-BIPQ - Total	-0.53	78	< 0.001	< 0.001	Yes	Yes
B-WHOQOL-BREF - Physical	B-BIPQ - Treatment control	0.13	84	0.23629	0.33691	No	No
B-WHOQOL-BREF - Physical	B-WHOQOL-BREF - Total	0.58	87	< 0.001	< 0.001	Yes	Yes
B-WHOQOL-BREF - Psychological	B-BIPQ - Coherence	0.15	84	0.16741	0.25111	No	No
B-WHOQOL-BREF - Psychological	B-BIPQ - Consequences	-0.42	84	< 0.001	< 0.001	Yes	Yes
B-WHOQOL-BREF - Psychological	B-BIPQ - Emotional representation	-0.42	84	< 0.001	< 0.001	Yes	Yes
B-WHOQOL-BREF - Psychological	B-BIPQ - Identity	-0.35	84	< 0.001	0.00230	Yes	Yes
B-WHOQOL-BREF - Psychological	B-BIPQ - Illness concern	-0.39	83	< 0.001	< 0.001	Yes	Yes
B-WHOQOL-BREF - Psychological	B-BIPQ - Personal control	0.37	83	< 0.001	0.00136	Yes	Yes
B-WHOQOL-BREF - Psychological	B-BIPQ - Timeline	-0.34	79	0.00227	0.00526	Yes	Yes
B-WHOQOL-BREF - Psychological	B-BIPQ - Total	-0.55	78	< 0.001	< 0.001	Yes	Yes
B-WHOQOL-BREF - Psychological	B-BIPQ - Treatment control	0.29	84	0.00679	0.01383	Yes	Yes
B-WHOQOL-BREF - Psychological	B-WHOQOL-BREF - Physical	0.59	87	< 0.001	< 0.001	Yes	Yes
B-WHOQOL-BREF	B-WHOQOL-	0.43	87	< 0.001	< 0.001	Yes	Yes

Variable 1	Variable 2	Spearman rho	Pairwise N	Unadjusted p	FDR-adjusted p	p < .05	FDR p < .05
- Psychological	BREF - Total						
B-WHOQOL-BREF - Social	B-BIPQ - Coherence	0.10	83	0.38757	0.48376	No	No
B-WHOQOL-BREF - Social	B-BIPQ - Consequences	-0.10	83	0.34595	0.45159	No	No
B-WHOQOL-BREF - Social	B-BIPQ - Emotional representation	-0.19	83	0.08166	0.13473	No	No
B-WHOQOL-BREF - Social	B-BIPQ - Identity	-0.06	83	0.61427	0.66905	No	No
B-WHOQOL-BREF - Social	B-BIPQ - Illness concern	-0.19	82	0.09541	0.15009	No	No
B-WHOQOL-BREF - Social	B-BIPQ - Personal control	0.01	82	0.91538	0.92077	No	No
B-WHOQOL-BREF - Social	B-BIPQ - Timeline	-0.32	78	0.00383	0.00830	Yes	Yes
B-WHOQOL-BREF - Social	B-BIPQ - Total	-0.20	77	0.08194	0.13473	No	No
B-WHOQOL-BREF - Social	B-BIPQ - Treatment control	0.04	83	0.75143	0.77406	No	No
B-WHOQOL-BREF - Social	B-WHOQOL-BREF - Physical	0.38	86	< 0.001	< 0.001	Yes	Yes
B-WHOQOL-BREF - Social	B-WHOQOL-BREF - Psychological	0.40	86	< 0.001	< 0.001	Yes	Yes
B-WHOQOL-BREF - Social	B-WHOQOL-BREF - Total	0.23	86	0.03193	0.05572	Yes	No
B-WHOQOL-BREF - Total	B-BIPQ - Coherence	0.08	84	0.48991	0.55479	No	No
B-WHOQOL-BREF - Total	B-BIPQ - Consequences	-0.50	84	< 0.001	< 0.001	Yes	Yes
B-WHOQOL-BREF - Total	B-BIPQ - Emotional representation	-0.29	84	0.00768	0.01528	Yes	Yes
B-WHOQOL-BREF - Total	B-BIPQ - Identity	-0.50	84	< 0.001	< 0.001	Yes	Yes
B-WHOQOL-BREF - Total	B-BIPQ - Illness concern	-0.33	83	0.00247	0.00556	Yes	Yes
B-WHOQOL-BREF - Total	B-BIPQ - Personal control	0.34	83	0.00163	0.00382	Yes	Yes
B-WHOQOL-BREF	B-BIPQ -	-0.26	79	0.01834	0.03337	Yes	Yes

Variable 1	Variable 2	Spearman rho	Pairwise N	Unadjusted p	FDR-adjusted p	p < .05	FDR p < .05
- Total	Timeline						
B-WHOQOL-BREF - Total	B-BIPQ - Total	-0.47	78	< 0.001	< 0.001	Yes	Yes
B-WHOQOL-BREF - Total	B-BIPQ - Treatment control	0.18	84	0.10379	0.16135	No	No

Table S3: Overview of the supplementary correlation heatmaps.

Figure	Sample	Variant	Interpretation
Supplementary Figure 1	F2	All correlations	All Spearman rho values are displayed
Supplementary Figure 2	F2	All correlations + FDR correction	All Spearman rho values are displayed; an empty cell marks correlations with FDR-adjusted p >= 0.05
Supplementary Figure 3	F3	All correlations	All Spearman rho values are displayed
Supplementary Figure 4	F3	All correlations + FDR correction	All Spearman rho values are displayed; an empty cell marks correlations with FDR-adjusted p >= 0.05

Figure S1: F2 Spearman correlation heatmap, including non-significant correlations (p > 0.05)

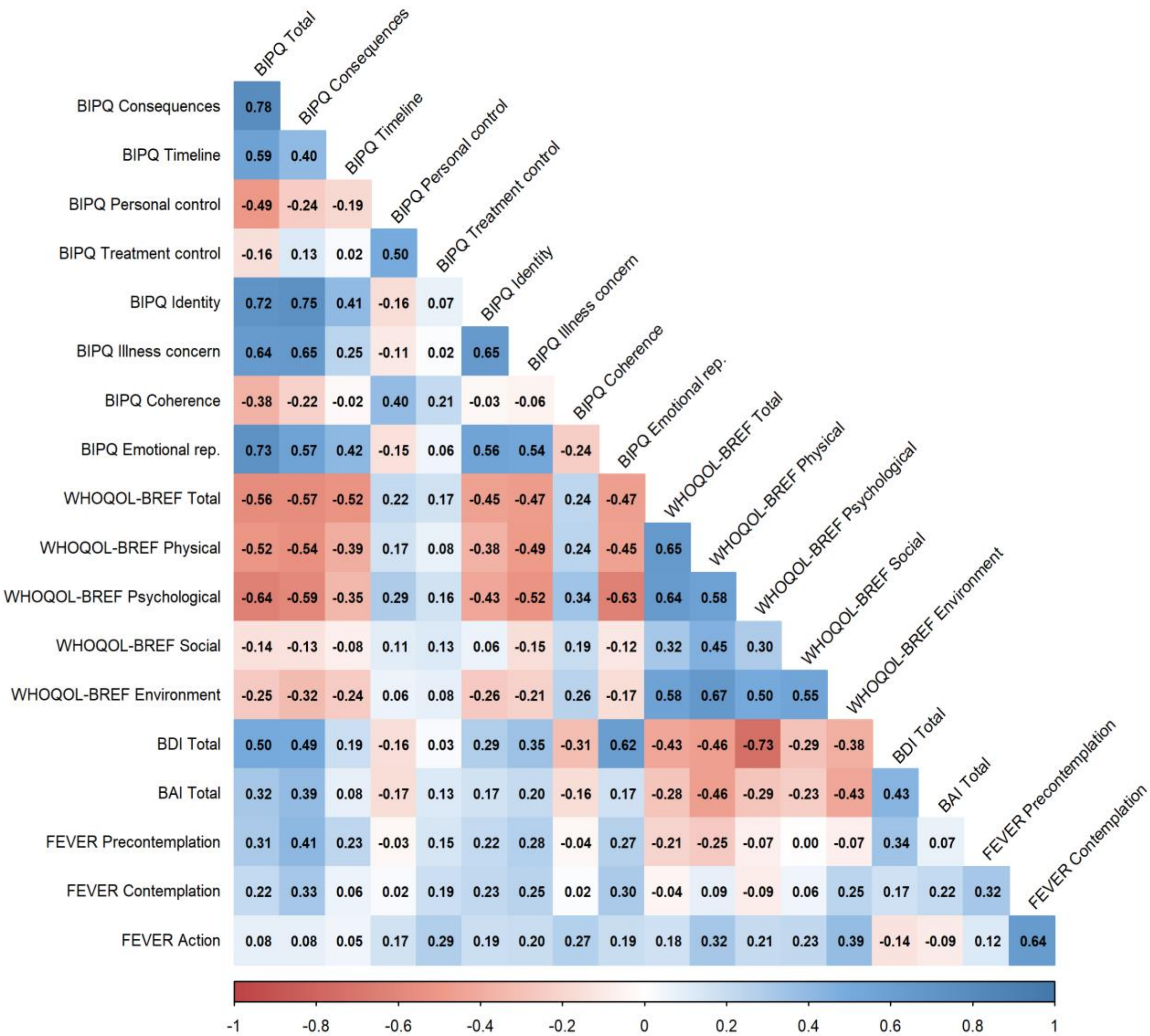

Figure S2: F2 Spearman correlation heatmap with FDR correction

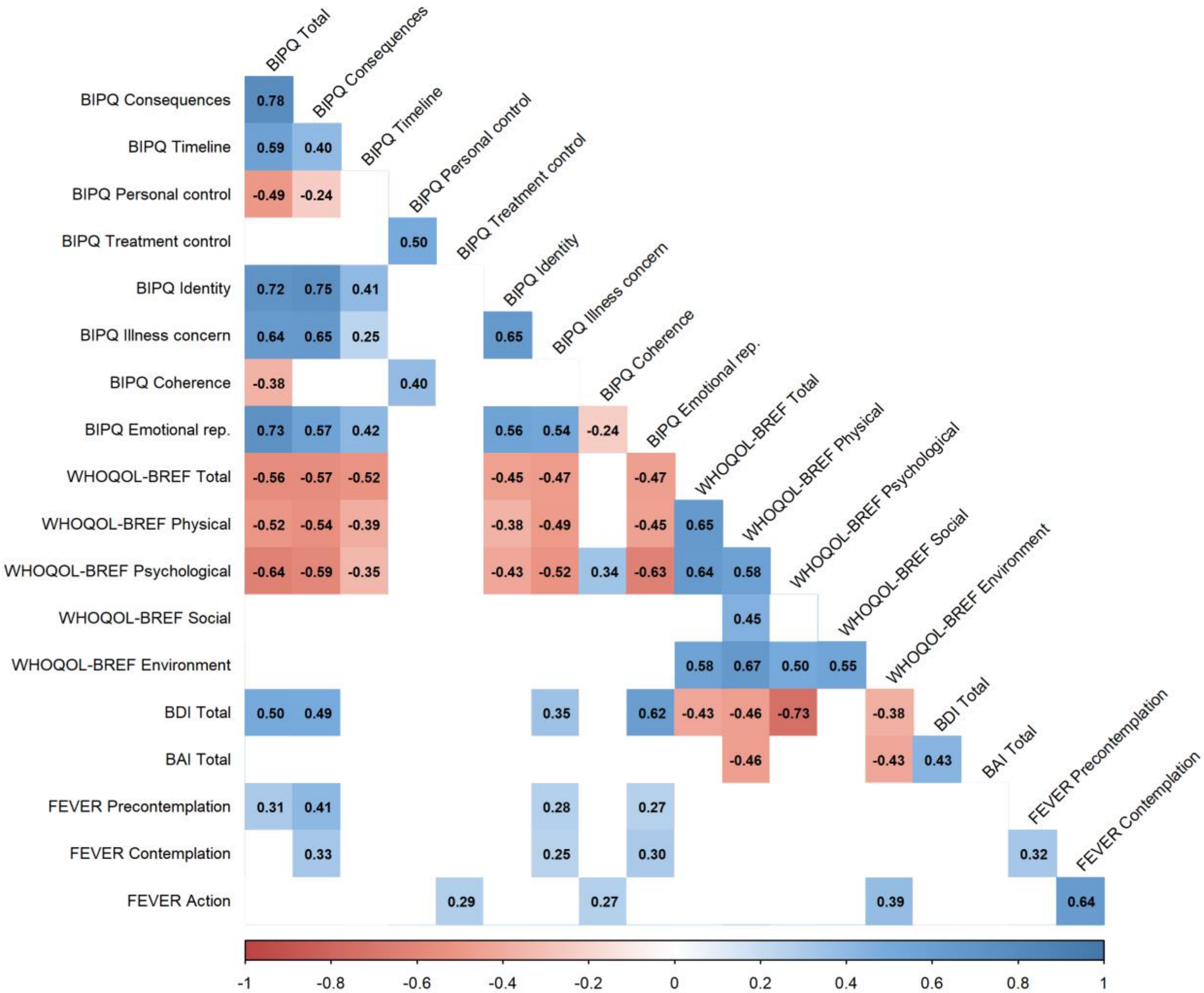

Note. Correlations that are not statistically significant after Benjamini-Hochberg FDR correction (adjusted p >= 0.05) are not shown.

Figure S3: F3 Spearman correlation heatmap, including non-significant correlations ($p > 0.05$)

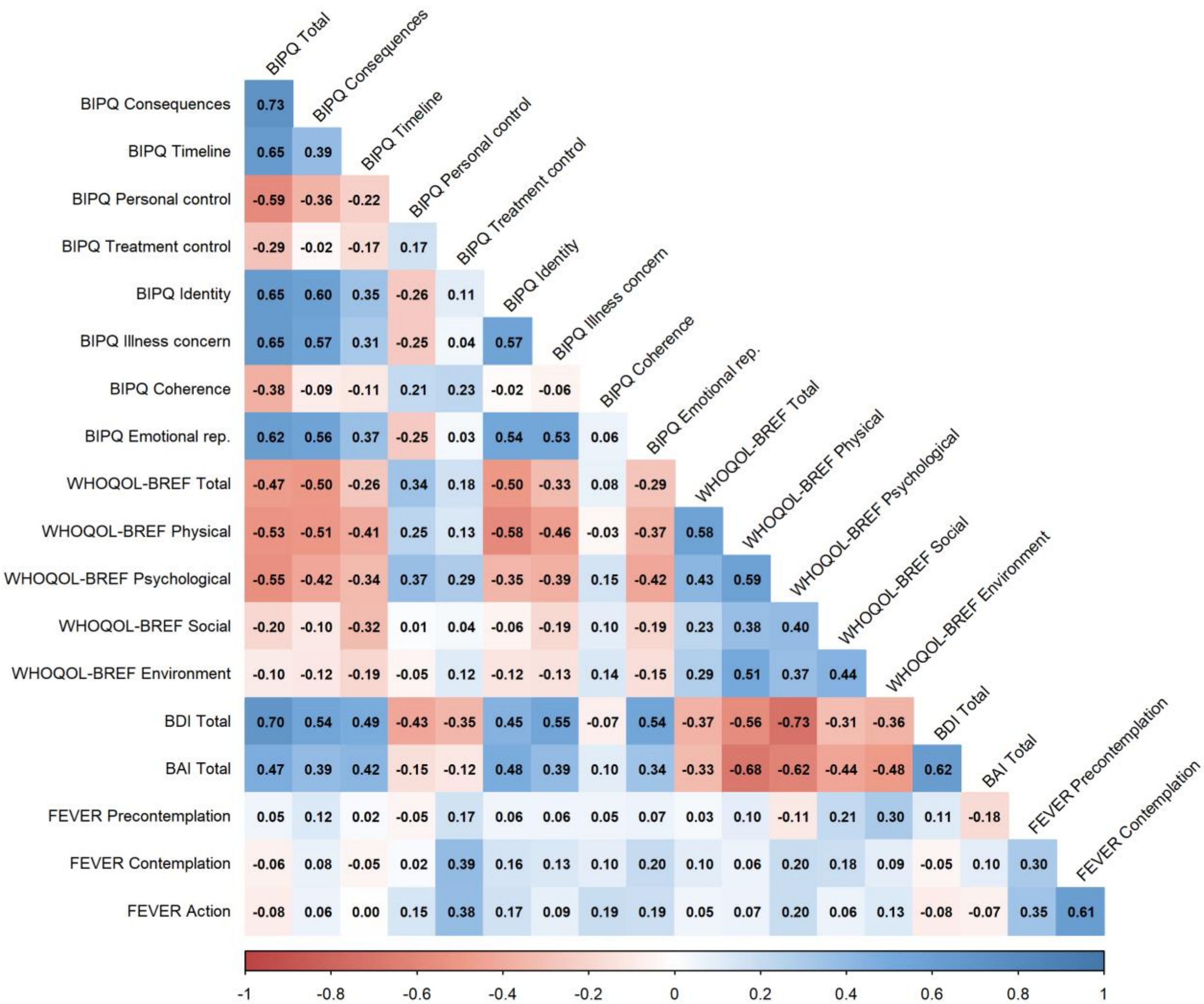

Figure S4: F3 Spearman correlation heatmap with FDR correction

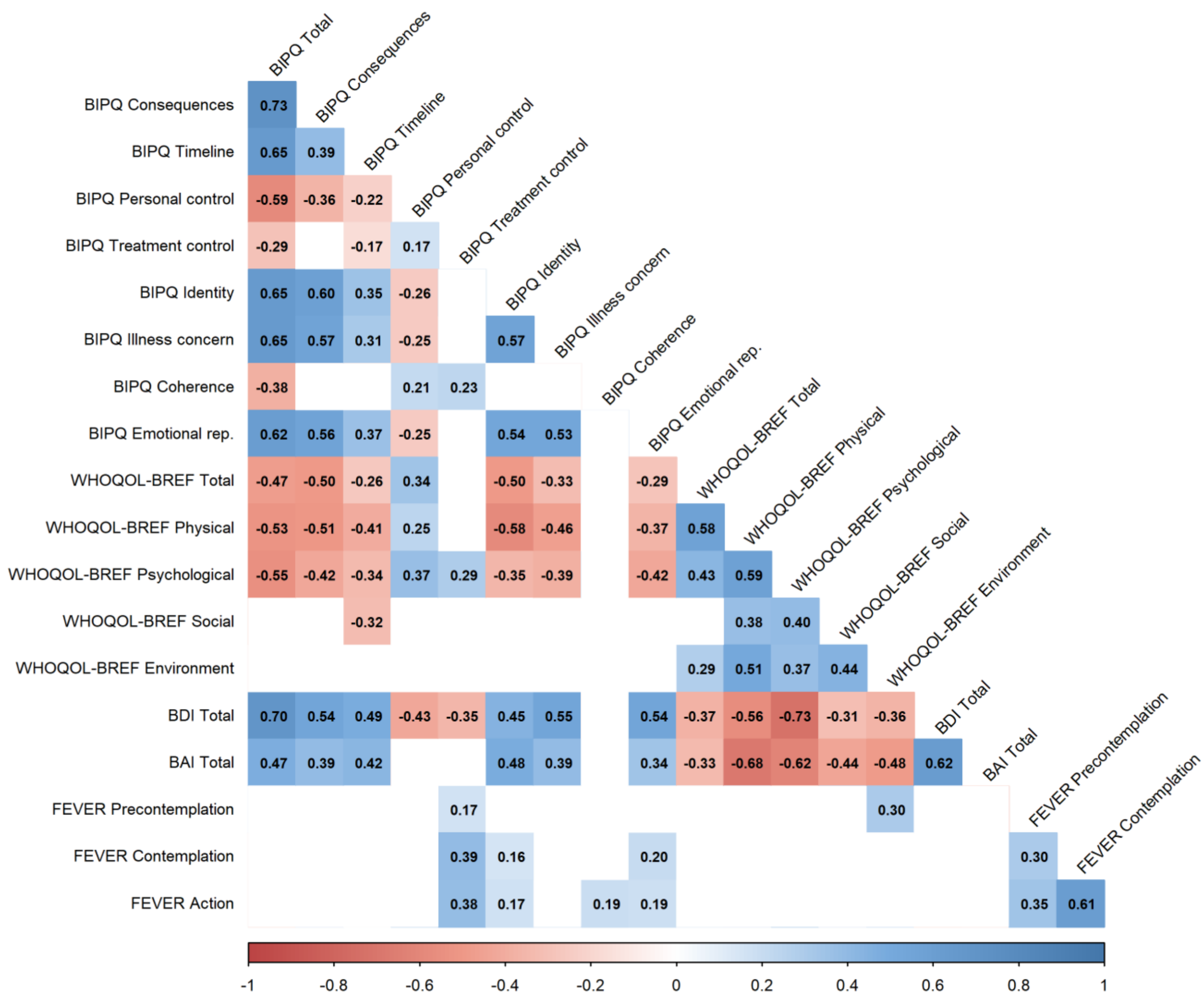

Correlations that are not statistically significant after Benjamini-Hochberg FDR correction (adjusted p >= 0.05) are not shown
